# Genome-Wide Interaction Study of Dietary Intake and Colorectal Cancer Risk in the UK Biobank

**DOI:** 10.1001/jamanetworkopen.2024.0465

**Published:** 2024-02-27

**Authors:** Tung Hoang, Sooyoung Cho, Ji-Yeob Choi, Daehee Kang, Aesun Shin

**Affiliations:** 1 Department of Preventive Medicine, Seoul National University College of Medicine, Seoul, Korea; 2 Integrated Major in Innovative Medical Science, Seoul National University Graduate School, Seoul, Korea; 3 Genomic Medicine Institute, Medical Research Center, Seoul National University, Seoul, Korea; 4 Department of Biomedical Sciences, Seoul National University Graduate School, Seoul, Korea; 5 BK21plus Biomedical Science Project, Seoul National University College of Medicine, Seoul, Korea; 6 Institute of Health Policy and Management, Medical Research Center, Seoul National University, Seoul, Korea; 7 Cancer Research Institute, Seoul National University, Seoul, Korea

## Abstract

**Importance:**

Candidate gene analysis approaches have shown that colorectal cancer (CRC) risk attributable to diet may differ according to genotype. A genome-wide approach further allows for the exploration of underlying pathways for associations between diet and CRC risk across the genome.

**Objectives:**

To identify genetic variants that modify diet-CRC associations and to further explore the underlying pathways in the cause of CRC.

**Design, Setting, and Participants:**

This nested case-control study used data on White British participants from the prospective cohort UK Biobank. Participants were recruited between March 13, 2006, and October 1, 2010, and data were censored June 25, 2021.

**Exposures:**

The average frequency intake of 11 dietary factors in the year preceding baseline was obtained via a touchscreen questionnaire. After quality control for more than 93 million variants of imputed genetic data, 4 122 345 variants remained.

**Main Outcomes and Measures:**

Colorectal cancer cases were identified according to the *International Statistical Classification of Diseases and Related Health Problems, Tenth Revision*. Genome-wide interaction analysis was performed to test interactions between dietary factors and variants using a conditional logistic regression model. Summary statistics of interactions at the variant level were used to calculate empirical *P* values for interactions at gene and gene-set levels in gene-based and gene-set enrichment analyses.

**Results:**

A total of 4686 participants with CRC (mean [SD] age, 60.7 [6.6] years; 2707 men [57.8%]) received a new diagnosis during a median of 12.4 years (IQR, 11.6-13.1 years) of follow-up. Once a case was detected, 3 matched controls were identified, for a total of 14 058 controls (mean [SD] age, 60.4 [6.6] years; 8121 men [57.8%]). A total of 324 variants were identified that interacted with diet consumption at the suggestive threshold (*P* < 1 × 10^−5^). In gene-based analysis, aggregation of multiple *EPDR1* gene variants was found to interact with fish intake regarding CRC risk. Furthermore, gene-set enrichment analysis found that several sets of protein-coding genes, which were overrepresented with particular functions and pathways, interacted with the consumption of milk (*ART*), cheese (*OR*), tea (*KRT*), and alcohol (*PRM* and *TNP*).

**Conclusions and Relevance:**

In this nested case-control study, the risk of CRC associated with fish intake was modified by multiple single-nucleotide polymorphisms of the *EPDR1* gene. The findings further suggested possible functions and pathways that might link the consumption of milk, cheese, tea, and alcohol with CRC development.

## Key Points

**Question:**

Which variants and genes modify the association of dietary intake with colorectal cancer (CRC) risk, and what are the underlying pathways for diet-CRC associations?

**Findings:**

In this nested case-control study including 4686 patients with incident CRC and 14 058 matched controls, 324 variants suggestively interacted with 11 dietary factors, and multiple variants of *EPDR1* were found to interact with fish intake on CRC risk. Several pathways were detected for the association between milk, cheese, tea, and alcohol consumption and CRC risk.

**Meaning:**

The findings of this study support evidence for possible pathways involved in the association between diet and CRC.

## Teaser

This nested case-control study uses data from the UK Biobank to identify genetic variants that modify associations between diet and colorectal cancer.

## Introduction

Colorectal cancer (CRC) is the second most commonly occurring cancer (519.8 thousand cases, 12.9% of all cancer sites) among European countries.^1^ In the UK, an estimated 95.3 thousand men and 65.9 thousand women received a diagnosis of CRC in 2020, ranking as the second and third highest cancer incidence, respectively.^1^

Taking results from the World Cancer Research Fund/American Institute for Cancer Research (WCRF/AICR) report in 2017,^2^ a recent umbrella review updated pooled estimates of foods and beverages for their associations with cancer, including CRC incidence.^3^ Red meat, processed meat, and alcohol consumption were associated with an elevated risk of CRC, whereas intake of fish, milk, fruit, and vegetables was found to be inversely associated with CRC risk.^2,3^ Pooled estimates for poultry, cheese, coffee, and tea did not show significant associations with CRC risk.^2,3^ In a recent work, we reported the association of genetic markers with food and beverage intake, with the explained proportions due to genetic variations ranging between 3.5% and 10.5%.^4^ Genetic factors may thus have an association with dietary habits, potentially leading to varying levels of CRC risk. Variations in genes involved in the metabolism of dietary factors have been shown to modify the association of foods with CRC risk.^5^ For instance, variations in the metabolism of carcinogenic substances produced when meat is cooked at high temperatures play an important role in CRC and modify the association of red and processed meat with CRC risk.^6^ The study also found inverse associations between cruciferous vegetables and CRC among individuals with a deficiency or intermediate levels of the enzyme involved in metabolism of isothiocyanates, which is abundant in cruciferous vegetables.^7,8,9^ Based on prior knowledge, a candidate gene approach that focused on genes involved in the metabolism of dietary intake has been implemented.^5^ As costs have decreased and technology has improved, genome-wide association studies have determined several genetic susceptibility loci for CRC,^5,10,11^ allowing for gene-diet interactions to be assessed across the genome.

Furthermore, dietary factors may also modulate gene expression in the pathogenesis of CRC directly, through their metabolites or by activating various signaling molecules of complex metabolic pathways.^12,13,14^ To date, the role of nutritional factors in several carcinogenesis pathways has been explored, such as DNA synthesis and epigenetic control of cell proliferation via folate-mediated one-carbon metabolism, DNA damage by free radicals, phase 1 and phase 2 enzymes, polynucleotide repair, and tumor promotion.^15^ Although possible biological mechanisms have been proposed to explain the association of dietary intake with CRC,^16^ specific genes and pathways that may be involved in the association between diet and CRC have not been documented.

In this study, by obtaining recently revealed imputed genetic data and CRC incident cases, we performed a gene-diet interaction analysis to identify genetic variants across the genome that interact with dietary factors in the association with CRC risk. However, the interaction between dietary factors and a single variant may not be well detected due to its weak effects. Therefore, we extended our investigation to multiple genetic markers in a gene-based test of gene-diet interactions. In addition, findings from our gene-set analyses might also be interpreted as indicating a cause of CRC by revealing gene sets involved in CRC tumorigenesis and modifying the association of diet.

## Methods

The UK Biobank study^17^ received ethical approval from the North West Multi-center Research Ethics Committee. Written informed consent was obtained from all participants before enrollment in the study. The present nested case-control cohort study was conducted using data from UK Biobank under application number 94695. The study protocol was approved by the institutional review board of Seoul National University. Reporting of this study followed the Strengthening the Reporting of Observational Studies in Epidemiology (STROBE) reporting guideline for cohort studies and the Strengthening the Reporting of Genetic Association Studies (STREGA) reporting guideline for genetic association studies.

### Study Population and Design

Details of the cohort design and procedures have been published previously.^18,19,20^ The overall UK Biobank sample consists of 487 181 participants, who were recruited from 22 assessment centers between March 13, 2006, and October 1, 2010, with both genetic and phenotypic data. In addition, we removed 367 individuals with self-reported vs genetically inferred sex mismatches and 651 individuals with putative sex chromosome aneuploidy. Given that disparities in diet consumption according to different racial and ethnic groups due to cultural knowledge and food-related skills^21,22^ can result in different amounts of intake that may be associated with the development of CRC, we restricted our analyses to White British individuals only. Accordingly, we removed 78 378 individuals who did not report their race or ethnicity or who were not genetically defined as having a White racial or ethnic background (including British, Irish, any other White background, and White). We excluded 34 078 individuals with prevalent cancer at recruitment and 11 individuals who withdrew from the study, with 374 004 participants remaining.

**Figure 1.**
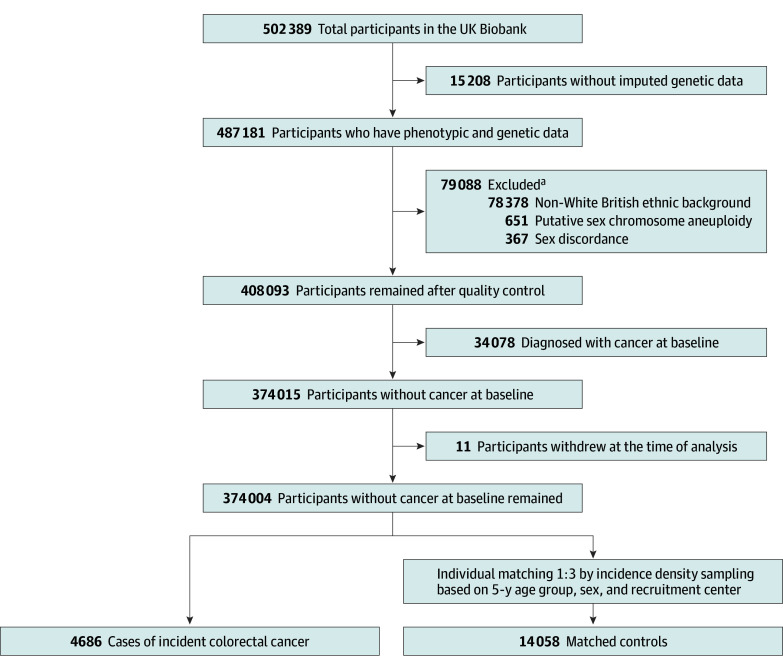
Flow Diagram of Case and Control Selection in the UK Biobank ^a^Numbers total more than 79 088 because of overlap among the categories.

During a median follow-up of 12.4 years (IQR, 11.6-13.1 years), 4686 patients received a new diagnosis of CRC. At the same time that each incident CRC case was detected, 3 controls were matched by age group at enrollment by 5 years, sex, and recruitment center using an incidence density sampling method (Figure 1).^23^ In the incidence density sampling approach, controls who were selected could become cases at a later time during the follow-up (463 participants). This approach differs from the case-control study design that uses a cumulative density sampling method to select controls from individuals remaining at risk after closing the study. The control series therefore provide an estimate of the proportion of the total person-time for exposed and unexposed cohorts among the source population.

### Dietary Intake Assessment and Genotyping Data

Information on average dietary intake in the preceding year was obtained via a touchscreen food frequency questionnaire.^24^ We included red meat, processed meat, poultry, total fish, milk, cheese, total fruit, total vegetables, coffee, tea, and alcohol in the final analysis. Details on the definition and calculation of each dietary factor were available in our previous work^4^ and in the eMethods in Supplement 1.

The quality control process for imputed genotyping data was available elsewhere.^4^ We further excluded single-nucleotide polymorphisms (SNPs) with a low imputation score (<0.3), high missingness (>0.05), low minor allele frequency (<0.05), and deviation from the expected Hardy-Weinberg equilibrium (*P* < 1 × 10^−6^),^25^ leaving 4 122 345 variants for the final genome-wide interaction (GWI) analysis (eMethods in Supplement 1).

### Outcome Ascertainment

Participants were followed up from baseline recruitment to the occurrence of CRC, loss to follow-up, death, or June 25, 2021, whichever was first. Colorectal cancer cases were identified according to the *International Statistical Classification of Diseases and Related Health Problems, Tenth Revision*, and CRC was coded as C18 to C20.

### Statistical Analysis

General characteristics of the participants with CRC and matched controls were compared using a Cochran-Mantel-Haenszel χ^2^ test for count data. We first sought to elucidate the association between dietary intake and CRC risk in a nested case-control design. The association was analyzed with a conditional logistic model to calculate odds ratios (ORs) and 95% CIs.

We performed a GWI analysis for 11 dietary phenotypes to identify loci that modify the association of diet consumption with CRC risk using a conditional logistic model. Using MAGMA (Multi-Marker Analysis of Genomic Annotation) in web-based FUMA (Functional Mapping and Annotation),^26^ summary statistics were then used as input in gene-based and gene-set analyses of identifying genes that multiple SNPs tended to converge and overrepresented pathways (eMethods in Supplement 1). Genotype data were handled in plink 2.0^27^ and entered into the R program, version 4.2.2 (R Project for Statistical Computing)^28^ for interaction analyses. Gene-based and gene-set analyses were performed using a web-based FUMA tool.^26^ To further interpret significant findings of gene-diet interactions, stratification analysis by genotypes was performed using 370 004 participants without cancer at baseline (eMethods in Supplement 1). A 2-sided *P* < 2.77 × 10^-6^ was deemed to be significant in gene-based analysis. We considered a Benjamini-Hochberg–adjusted 2-sided *P* < .05 as significant in gene-set enrichment analysis.

## Results

### Characteristics of the Study Population

Table 1 summarizes the demographic characteristics of 4686 participants (mean [SD] age, 60.7 [6.6] years; 2707 men [57.8%]) with incident CRC and 14 058 matched controls (mean [SD] age, 60.4 [6.6] years; 8121 men [57.8%]) in the final analysis. Distributions of matching factors, including 5-year age group, sex, and recruitment center, were similar between the 2 groups. Compared with the controls, the cases were more likely to be past or current smokers (past, 2037 [43.5%] vs 5525 [39.3%]; current, 457 [9.8%] vs 1296 [9.2%]; *P* < .001), more frequently drank alcohol (daily or more often, 1243 [26.5%] vs 3353 [23.9%]; *P* = .002), and were more likely to have obesity (1278 [27.3%] vs 3504 [24.9%]; *P* < .001). Among 18 281 participants included in the main analysis, dietary data at more than 1 follow-up visit were available for 10.2% of participants (n = 1867).

**Table 1.** Characteristics of Study Participants in a Nested Case-Control From the UK Biobank

Characteristic	Participants, No. (%)		*P* value^a^
	CRC cases (n = 4686)	Matched controls (n = 14 058)	
Age, y			
40 to <45	121 (2.6)	363 (2.6)	NA
45 to <50	247 (5.3)	741 (5.3)	
50 to <55	487 (10.4)	1461 (10.4)	
55 to <60	777 (16.6)	2331 (16.6)	
60 to <65	1449 (30.9)	4347 (30.9)	
65 to <70	1555 (33.2)	4665 (33.2)	
70 to <75	50 (1.1)	150 (1.1)	
Sex			
Women	1979 (42.2)	5937 (42.2)	NA
Men	2707 (57.8)	8121 (57.8)	
UK Biobank recruitment center			
Barts	75 (1.6)	225 (1.6)	NA
Birmingham	181 (3.9)	543 (3.9)	
Bristol	404 (8.6)	1212 (8.6)	
Bury	316 (6.7)	948 (6.7)	
Cardiff	132 (2.8)	396 (2.8)	
Croydon	177 (3.8)	531 (3.8)	
Edinburgh	224 (4.8)	672 (4.8)	
Glasgow	220 (4.7)	660 (4.7)	
Hounslow	179 (3.8)	537 (3.8)	
Leeds	402 (8.6)	1206 (8.6)	
Liverpool	311 (6.6)	933 (6.6)	
Manchester	144 (3.1)	432 (3.1)	
Middlesbrough	187 (4.0)	561 (4.0)	
Newcastle	402 (8.6)	1206 (8.6)	
Nottingham	327 (7.0)	981 (7.0)	
Oxford	170 (3.6)	510 (3.6)	
Reading	303 (6.5)	909 (6.5)	
Sheffield	292 (6.2)	876 (6.2)	
Stockport	8 (0.2)	24 (0.2)	
Stoke	216 (4.6)	648 (4.6)	
Swansea	13 (0.3)	39 (0.3)	
Wrexham	3 (0.1)	9 (0.1)	
Smoking status			
Never	2178 (46.5)	7186 (51.1)	<.001
Former	2037 (43.5)	5525 (39.3)	
Current	457 (9.8)	1296 (9.2)	
Missing	14 (0.3)	51 (0.4)	
Alcohol consumption			
Never or rarely	778 (16.6)	2378 (16.9)	.002
Once a month to twice a week	1561 (33.3)	4956 (35.3)	
3-4 Times/wk	1097 (23.4)	3360 (23.9)	
Daily or more	1243 (26.5)	3353 (23.9)	
Missing	7 (0.1)	11 (0.1)	
BMI			
<25.0	1240 (26.5)	4217 (30.0)	<.001
25.0 to <30.0	2151 (45.9)	6293 (44.8)	
≥30.0	1278 (27.3)	3504 (24.9)	
Missing	17 (0.4)	42 (0.3)	
Moderate or vigorous activity			
Insufficient	1769 (37.8)	5054 (36.0)	.05
Sufficient	2017 (43.0)	6317 (44.9)	
Missing	900 (19.2)	2687 (19.1)	

Abbreviations: BMI, body mass index (calculated as weight in kilograms divided by height in meters squared); CRC, colorectal cancer; NA, not applicable.

^a^ Calculated from a Cochran-Mantel-Haenszel χ^2^ test.

### Associations Between Dietary Intake and Colorectal Cancer Risk

eTable 1 in Supplement 1 shows the association of each dietary factor with CRC risk. Compared with individuals who consumed red meat fewer than 2 times per week and processed meat fewer than 1 time per week, those who consumed red meat 3 or more times per week and processed meat 2 or more times per week were more likely to have higher risks of CRC (OR, 1.16 [95% CI, 1.07-1.27] and 1.16 [95% CI, 1.07-1.26], respectively) after adjusting for confounding factors. In addition, participants who consumed alcohol 3 or more times per week were shown to have an increased risk of CRC (OR, 1.10 [95% CI, 1.01-1.20]) compared with those who consumed alcohol fewer than 1 time per week. Protective associations against CRC were observed for fruit intake (OR, 0.90 [95% CI, 0.82-0.99]) among participants who consumed 4 or more servings per day of fruit compared with those who consumed fewer than 2 servings per day.

### Genome-Wide Gene-Diet Interaction Analysis

eFigure 1A to F and eFigure 2A to E in Supplement 1 show the Manhattan plot for the negative logarithm of *P* values of gene-diet interaction terms obtained from genome-wide gene-diet interaction analysis. We identified a total of 324 SNPs that suggestively interact with red meat (n = 13), processed meat (n = 12), poultry (n = 6), fish (n = 34), milk (n = 31), cheese (n = 58), fruits (n = 40), coffee (n = 80), tea (n = 17), and alcohol (n = 33) consumption (eTable 2 in Supplement 1). None of the genetic variants reached the genome-wide significance level.

### Gene-Based Analysis

**Figure 2.**
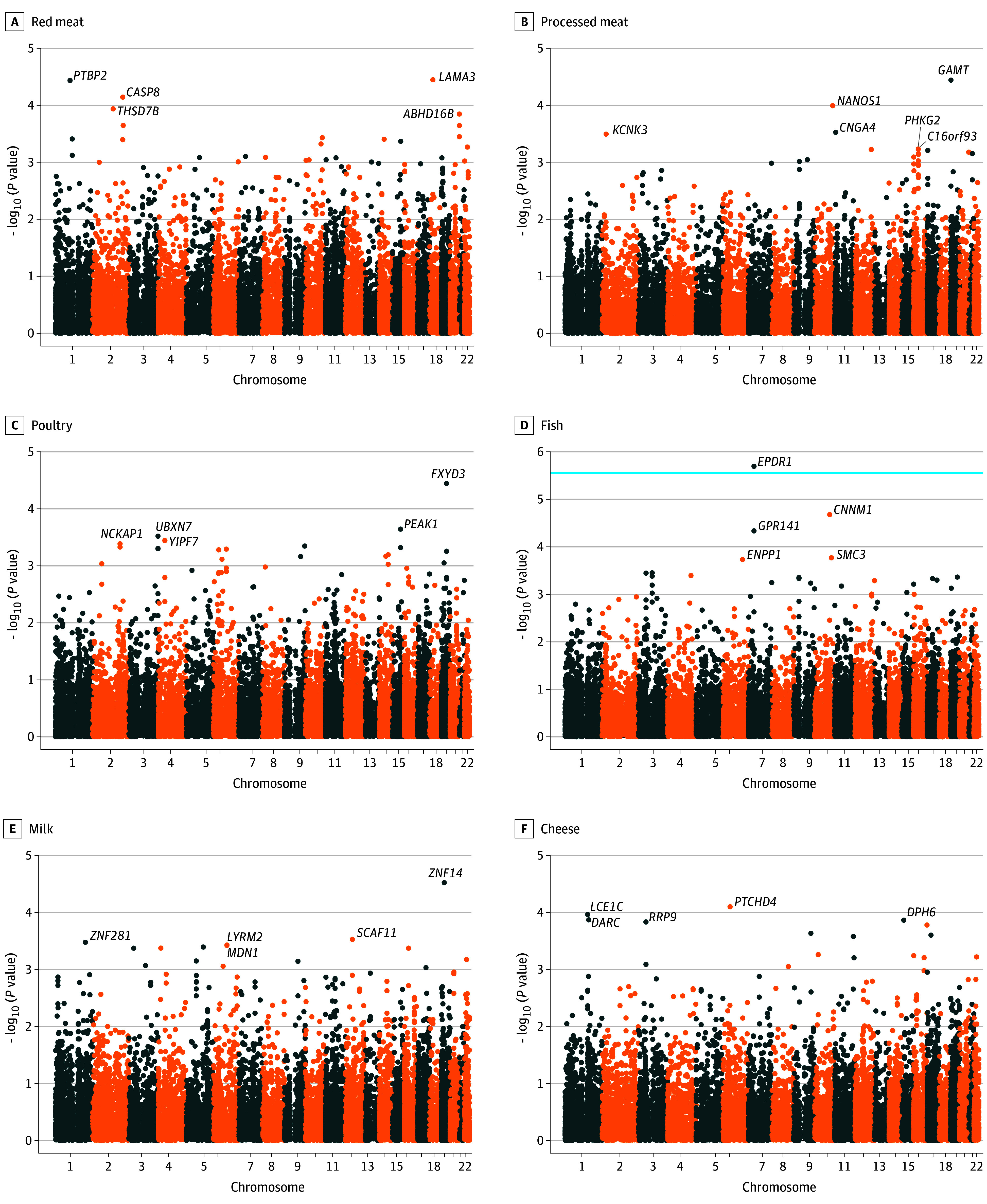
Manhattan Plot of Interacted Genes Identified in the Marker Analysis of Genomic Annotation Gene-Based Analysis The light blue horizontal line indicates threshold for level of significance (*P* < 2.77 × 10^–6^). The top 5 significant genes are labeled.

**Figure 3.**
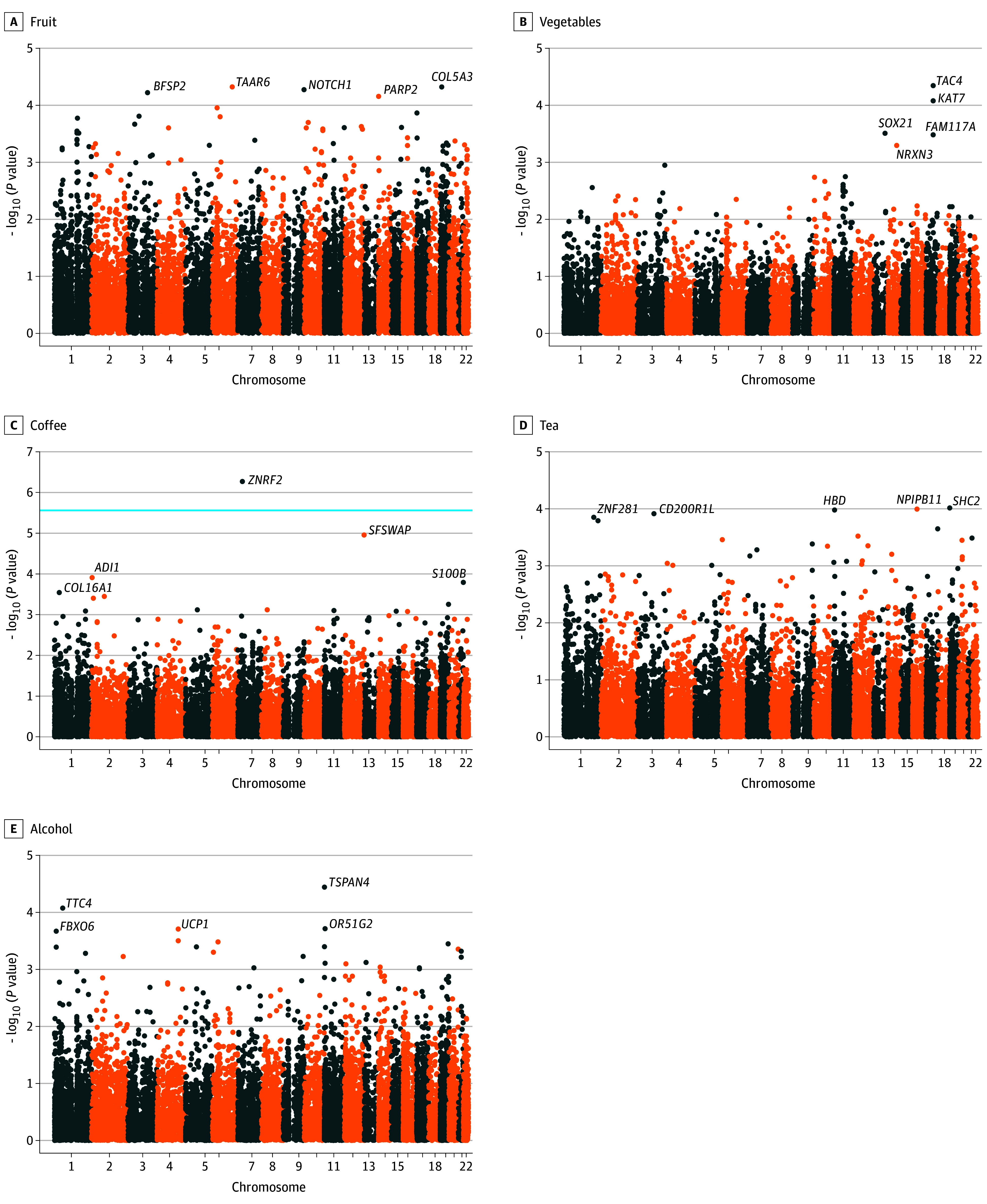
Manhattan Plot of Interacted Genes Identified in the Marker Analysis of Genomic Annotation Gene-Based Analysis The light blue horizontal line indicates threshold for level of significance (*P* < 2.77 × 10^–6^). The top 5 significant genes are labeled.

Figure 2A to F and Figure 3A to E show the Manhattan plot of the gene-based test implemented in MAGMA using summary statistics from GWI analysis. The top 5 genes identified by SNP-diet interactions were presented for all dietary factors. Among them, *EPDR1* (Ensembl ENSG00000086289) and *ZNRF2* (Ensembl ENSG00000180233) reached a significant threshold in their interaction with fish and coffee consumption, respectively. However, *EPDR1* was observed with multiple SNPs (46 SNPs, minor allele frequency between 0.316 and 0.442; eTable 3 in Supplement 1), whereas *ZNRF2* contained 1 SNP only (chromosome 7: rs34294791).

### Gene-Set Enrichment Analysis

In gene-set enrichment analysis, several genes that most strongly interacted with the consumption of milk (*ART*), cheese (*OR*), tea (*KRT*), and alcohol (*PRM* and *TNP*) were found to be overrepresented within particular pathways (Table 2). In particular, we found that the gene sets interacting with milk intake mainly share arginine adenosine diphosphate (ADP)–ribosyltransferase (*ART*) activity with the *ART* gene family (eFigure 3 in Supplement 1). Gene sets interacting with cheese intake mainly share sensory perception activity via G protein–coupled receptors with the olfactory receptor (*OR*) gene family (eFigure 4 in Supplement 2). In addition, gene sets interacting with tea consumption were found to overlap with the keratin (*KRT*) gene family in sharing skin development–related processes (eFigure 5 in Supplement 1). Alcohol consumption interacted with gene sets that share biological pathways with protein-coding genes (*PRM1* [Ensembl ENSG00000175646], *PRM2* [Ensembl ENSG00000122304], *PRM3* [Ensembl ENSG00000178257], and *TNP2* [Ensembl ENSG00000178279]) involved in chromosome condensation and male infertility activities (eFigure 6 in Supplement 1).

**Table 2.** Significantly Enriched Gene Sets That Interacted With Dietary Intake

Food, source, and function	No. of genes in gene sets	Overlapping genes	*P* value	Adjusted *P* value
**Milk**				
GO molecular function				
NAD(P) + arginine ADP-ribosyltransferase activity	5	*ART5*, *ART1*	5.72 × 10^–6^	9.42 × 10^–3^
**Cheese**				
GO biological process				
Sensory perception of smell	394	*OR8D4*, *OR4D5*, *OR6T1*, *OR10S1*, *OR10G4*, *OR10G9*, *OR10G8*, *OR10G7*	1.01 × 10^–10^	7.85 × 10^–7^
Detection of chemical stimulus	447	*OR8D4*, *OR4D5*, *OR6T1*, *OR10S1*, *OR10G4*, *OR10G9*, *OR10G8*, *OR10G7*	2.76 × 10^–10^	9.72 × 10^–7^
Sensory perception of chemical stimulus	469	*OR8D4*, *OR4D5*, *OR6T1*, *OR10S1*, *OR10G4*, *OR10G9*, *OR10G8*, *OR10G7*	4.04 × 10^–10^	9.72 × 10^–7^
Detection of stimulus involved in sensory perception	482	*OR8D4*, *OR4D5*, *OR6T1*, *OR10S1*, *OR10G4*, *OR10G9*, *OR10G8*, *OR10G7*	5.02 × 10^–10^	9.72 × 10^–7^
Detection of stimulus involved in sensory perception	596	*OR8D4*, *OR4D5*, *OR6T1*, *OR10S1*, *OR10G4*, *OR10G9*, *OR10G8*, *OR10G7*	2.68 × 10^–9^	4.16 × 10^–6^
Sensory perception	916	*OR8D4*, *OR4D5*, *OR6T1*, *OR10S1*, *OR10G4*, *OR10G9*, *OR10G8*, *OR10G7*	7.72 × 10^–8^	9.97 × 10^–5^
G protein–coupled receptor signaling pathway	1172	*OR8D4*, *OR4D5*, *OR6T1*, *OR10S1*, *OR10G4*, *OR10G9*, *OR10G8*, *OR10G7*	5.17 × 10^–7^	5.72 × 10^–4^
Nervous system process	1429	*OR8D4*, *OR4D5*, *OR6T1*, *OR10S1*, *OR10G4*, *OR10G9*, *OR10G8*, *OR10G7*	2.34 × 10^–6^	2.27 × 10^–3^
Molecular function				
G protein–coupled receptor activity	768	*OR8D4*, *OR4D5*, *OR10S1*, *OR10G4*, *OR10G9*, *OR10G8*, *OR10G7*	5.29 × 10^–7^	9.38 × 10^–4^
Molecular transducer activity	1384	*OR8D4*, *OR4D5*, *OR6T1*, *OR10S1*, *OR10G4*, *OR10G9*, *OR10G8*, *OR10G7*	1.84 × 10^–6^	1.63 × 10^–3^
**Tea**				
GO biological process				
Intermediate filament organization	71	*KRT74*, *KRT72*, *KRT73*, *KRT2*	2.90 × 10^–9^	1.93 × 10^–5^
Keratinization	81	*KRT74*, *KRT72*, *KRT73*, *KRT2*	4.97 × 10^–9^	1.93 × 10^–5^
Intermediate filament-based process	90	*KRT74*, *KRT72*, *KRT73*, *KRT2*	7.62 × 10^–9^	1.97 × 10^–5^
Epidermal cell differentiation	169	*KRT74*, *KRT72*, *KRT73*, *KRT2*	9.72 × 10^–8^	1.88 × 10^–4^
Keratinocyte differentiation	232	*KRT74*, *KRT72*, *KRT73*, *KRT2*	3.47 × 10^–7^	5.37 × 10^–4^
Skin development	296	*KRT74*, *KRT72*, *KRT73*, *KRT2*	9.18 × 10^–7^	1.19 × 10^–3^
Epidermis development	360	*KRT74*, *KRT72*, *KRT73*, *KRT2*	2.01 × 10^–6^	2.22 × 10^–3^
Epithelial cell differentiation	693	*KRT74*, *KRT72*, *KRT73*, *KRT2*	2.70 × 10^–5^	2.61 × 10^–2^
Supramolecular fiber organization	770	*KRT74*, *KRT72*, *KRT73*, *KRT2*	4.09 × 10^–5^	3.52 × 10^–2^
GO cellular component				
Keratin filament	92	*KRT74*, *KRT72*, *KRT73*, *KRT2*	8.33 × 10^–9^	8.41 × 10^–6^
Intermediate filament	194	*KRT74*, *KRT72*, *KRT73*, *KRT2*	1.69 × 10^–7^	8.53 × 10^–5^
Intermediate filament cytoskeleton	231	*KRT74*, *KRT72*, *KRT73*, *KRT2*	3.41 × 10^–7^	1.15 × 10^–4^
Polymeric cytoskeletal fiber	726	*KRT74*, *KRT72*, *KRT73*, *KRT2*	3.24 × 10^–5^	8.17 × 10^–3^
Supramolecular polymer	971	*KRT74*, *KRT72*, *KRT73*, *KRT2*	1.02 × 10^–4^	2.05 × 10^–2^
GO molecular function				
Structural constituent of skin epidermis	36	*KRT74*, *KRT72*, *KRT73*, *KRT2*	1.77 × 10^–10^	3.13 × 10^–7^
Structural molecule activity	703	*KRT74*, *KRT72*, *KRT73*, *KRT2*	2.85 × 10^–5^	2.53 × 10^–2^
**Alcohol**				
GO biological process				
Chromosome condensation	43	*PRM3*, *PRM2*, *PRM1*	9.57 × 10^–7^	7.42 × 10^–3^
GO cellular component				
Nucleosome	87	*TNP2*, *PRM3*, *PRM2*, *PRM1*	5.52 × 10^–8^	5.57 × 10^–5^
DNA packaging complex	118	*TNP2*, *PRM3*, *PRM2*, *PRM1*	1.89 × 10^–7^	9.54 × 10^–5^
Protein-DNA complex	176	*TNP2*, *PRM3*, *PRM2*, *PRM1*	9.40 × 10^–7^	3.16 × 10^–4^
WikiPathway				
Male infertility	131	*PRM3*, *PRM2*, *PRM1*	2.78 × 10^–5^	2.04 × 10^–2^

Abbreviations: ADP, adenosine diphosphate; GO, gene ontology.

### Interpretation of Gene-Diet Interaction

Among 370 004 cancer-free individuals at baseline, a total of 330 651 individuals not missing any genotype data for 46 SNPs located in *EPDR1* were further included in the stratification analysis. The first principal component derived from the principal component analysis explained 94.2% variance of multiple variants in the *EPDR1* gene (eFigure 7 in Supplement 1). This pattern appeared to be associated with more reference alleles of rs2709110, rs2718059, rs1450847, rs35202238, rs1597550, and rs1376256 and fewer reference alleles of the remaining variants in the *EPDR1* gene (eTable 4 in Supplement 1).

Using the mean value of the principal component score as the cutoff, individuals were classified into a low-score group (n = 190 154; fewer reference alleles of rs2709110, rs2718059, rs1450847, rs35202238, rs1597550, and rs1376256 variants) and a high-score group (n = 140 497; more reference alleles of rs2709110, rs2718059, rs1450847, rs35202238, rs1597550, and rs1376256 variants). Accordingly, fish intake was not associated with CRC risk among those in the low-score group; however, consuming fish more than 2 times per week was associated with a decreased risk of CRC among individuals in the high-score group, with hazard ratios of 0.852 (95% CI, 0.746-0.972), 0.875 (95% CI, 0.766-0.998), and 0.851 (95% CI, 0.732-0.990) in the crude, sex-adjusted, and fully adjusted models, respectively (eTable 5 in Supplement 1).

## Discussion

In this nested case-control setting, dietary intake of red meat, processed meat, and alcohol was associated with an increased risk of CRC, while consumption of 4 or more servings of fruit per day was associated with a decreased risk of CRC. Genome-wide imputation analyses identified 324 SNPs that suggestively interacted with dietary factors. Although no variant was found to interact with dietary intake at the level of significance, gene-based analysis detected that gene-fish consumption interaction effects tended to congregate within *EPDR1*. Furthermore, our gene-set enrichment analysis identified biological pathways shared by interacting gene sets and previously known gene families and protein-coding genes (eFigure 8 in Supplement 1).

Our study found a significant association between CRC and consumption of red meat 3 or more times per week and processed meat 2 or more times per week. The International Agency for Research on Cancer classifies processed meat and alcohol as human carcinogens (group 1) and red meat as a probable carcinogen (group 2A).^29^ The WCRF/AICR also reported probable convincing evidence of carcinogenic associations of red meat, processed meat, and alcohol with increasing CRC risk.^30^ One study examined the association between dietary intake and CRC risk in a prospective cohort study design using UK Biobank data and reported an increased risk of CRC by 18%, 19%, and 24% in association with red meat, processed meat, and alcohol, respectively.^31^ Although there was still suggestive evidence of low fruit intake associated with CRC risk,^30^ our present study, which followed up with the participants for a longer period (12.4 years vs 5.7 years^31^), observed a protective association against CRC for frequent consumption of fruits.

Among the methods for detection of gene-environment interactions, the results from 2-step approaches may reveal significant interactions at the SNP level only.^32^ In the 2-step approach, a marginal test can be first implemented to screen for genetic variants associated with the outcome.^32,33^ Then, SNPs that pass the significance threshold in step 1 can be tested for their interaction term with environmental exposure to improve statistical power.^32,33,34^ However, there may still be a risk of missing potential gene-environment interactions when excluding many variants in the screening step. In contrast, the 1-step approach in our present study was able to generate the summary statistics from a GWI study^32^; thus, we may be unable to identify interactable genes in gene-based tests and functional annotations in gene-set enrichment analyses. By following the 1-step approach, we implemented the analysis in a nested case-control approach to reduce control selection bias and improve the temporal association between exposures and outcomes, although a novel method was developed to account for the case-control imbalance in a case-control study design.^35,36^ In addition, compared with the standard case-control setting, a nested case-control approach under a predefined cohort may reduce control selection bias and improve the temporal association between exposures and outcomes.^35^

To our knowledge, few studies to date have evaluated the interaction between genes and diets in the whole genome in CRC development.^10^ A pooled analysis from 9287 cases and 9117 controls from the Colon Cancer Family Registry and Genetics and Epidemiology of Colorectal Cancer Consortium tested the multiplicative interaction of approximately 2.7 million SNPs with main dietary factors. such as red meat, processed meat, fruits, vegetables, and fiber.^37^ Only a significant interaction between rs4143094 (*GATA3* [Ensembl ENSG00000107485]) and processed meat consumption was reported at genome-wide significance.^11^ Another study using data from 8058 cases and 8765 controls from the same consortium reported interactions between variants located at the 9q22.32-*HIATL1* (Ensembl ENSG00000148110) locus and alcohol consumption.^38^ In contrast, another investigation using UK Biobank data of 2652 cases and 10 608 controls did not detect any SNPs interacting with dietary factors of red meat, processed meat, fruits, vegetables, fiber, or calcium.^39^ In the present study, we extended our investigation to more than 4 million SNPs and a much greater number of incident cases in the nested case-control setting, and none of the variants reached the significance threshold.

Despite GWI study results of SNP-diet interaction associations across the genome, interpretation of particular genes in the interaction with dietary factors has not been facilitated. In the present study, our gene-based analysis results suggested that aggregation multiple 46 SNPs in *EPDR1* may interact with fish intake with regard to CRC risk. Possible links between fish consumption and CRC carcinogenesis have been introduced via the properties of omega-3 polyunsaturated fatty acids. In brief, eicosapentaenoic and docosahexaenoic acids can inhibit prostaglandin E2 production and secondary bile acids, thus exerting anti-inflammatory properties and altering proliferation and lipid metabolism of cancer cells.^40^ Similar properties have also been documented for *EPDR1*, which may hypothetically explain their interactions with CRC development. The *EPDR1* gene encodes a protein similar to ependymins, which play important roles in adhesion of neural cells by intracellular and extracellular mechanisms.^41,42^ Ependymins were first observed in fish brains, where they are involved in the formation of long-term memory and neuronal regeneration.^41^ To date, studies have demonstrated that *EPDR1* is upregulated in patients with CRC and might increase cell proliferation, cell migration, interaction with type I collagen fibrils, and invasiveness in CRC cell lines.^43^ Hypermethylation of *EPDR1* is also associated with node negativity and lower tumor stage with variations in *BRAF* (Ensembl ENSG00000157764) and *TGFβR2* (Ensembl ENSG00000163513).^44^ Moreover, comparative analysis of the secretomes of human brown and white adipocytes suggest a vital role for *EPDR1* in the development of thermogenesis during adipogenesis; thus, *EPDR1* may be involved in regulating human metabolism.^45^

Findings from our gene-set enrichment analysis further suggest possible biological and functional pathways for the association of dietary intake with CRC risk. We found that milk intake interacted with gene sets overrepresented in arginine ADP-ribosyltransferase activity. Among these genes, *ART1* (Ensembl ENSG00000129744) is known to promote proliferation, invasion, and metastasis in colon carcinoma, regulate glycolysis, and be involved in CRC angiogenesis via the *PI3K*-*Akt* pathway.^46,47^ This pathway is also linked to regulation of several human metabolism processes, including lipid and glucose metabolism.^48^ In addition, consumption of calcium in milk may interfere with lipid absorption by binding to fatty acids and bile acids in the gut.^49^ Regarding cheese intake, we observed its interaction with gene sets overrepresented in the detection of chemical stimuli involved in sensory perception. Among these genes, *OR10G4* (Ensembl ENSG00000254737) was associated with odorant perception of guaiacol (adjusted *P* < .001) in a high-throughput sequencing study of the whole olfactory receptor that extensively determined several genetic variations associated with different phenotypes.^50^ Guaiacol is one of the most important compounds that contribute to the “smokiness” of cheddar cheese, which is the most popular cheese in the US.^51^ Furthermore, we found that tea consumption interacted with gene sets overrepresented in keratinization, keratin filament, and intermediate filament pathways. These pathways are involved in the antiacne activity of epigallocatechin-3-gallate, which is a major polyphenol in green tea^52^ and observed to be enriched in onset of colon cancer.^53^ Previous studies suggested the association of long-term alcohol consumption with poor semen quality due to oxidative stress and genotoxicity on hormonal regulation and DNA integrity.^54^ In addition, *PRM1* is associated with proliferation, invasion, and migration of colon cancer cells,^55^ and male infertility also shares its genetic cause with cancer.^56^ These findings may support our finding of the interaction between alcohol consumption and gene sets overrepresented in the male infertility pathway.

Apart from gene-diet interaction analysis at the variant level, our gene-based and gene-set analyses further allowed us to evaluate significant interactions at higher levels. We did not detect any significant SNPs at the variant level due to weak effect problems. However, when considering multiple genetic markers together, we were able to detect significant joint associations of multiple markers located only in a specific *EPDR1* gene region. Considering multiple genes together, we further determined several functional and biological insights that may be involved in the link between diet and the development of CRC.

### Limitations

This study has some limitations. One limitation is the fact that we analyzed CRC risk using the dietary information measured only at a single time point, and we assumed that such dietary changes might not be associated with the overall gene-diet interactions. In addition to data collected at baseline (2006-2010), we obtained dietary information from 3 subsequent reassessment visits (2009-2013, since 2013, and since 2018). Among 18 281 participants included in the main analysis, dietary data at more than 1 follow-up visit were available for 10.2% of participants (n = 1867). The intake of processed meat, fruit, vegetables, and tea remained stable; however, participants were observed to significantly change their consumption of other dietary factors (eTable 6 in Supplement 1). Furthermore, there is also a limitation associated with the use of gene-based analyses. Empirical *P* values were computed for particular genes by using summary statistics obtained from GWI analyses. Thus, when combining the interactive associations of multiple variants, gene-based analyses were unable to confirm whether certain SNPs in genes may modify the association between dietary factors and CRC risk.^57^ Instead, our results suggested that individuals who carry more reference alleles of rs2709110, rs2718059, rs1450847, rs35202238, rs1597550, and rs1376256 showed an association between consumption of fish more than 2 times per week and reduction of CRC risk. Last, our findings are limited by a lack of validation in independent data and generalization to non-European populations.

## Conclusion

In this nested case-control study, we identified several SNPs that interacted with dietary intake at the suggestive threshold. When genetic variants congregated in certain genes, there were significant interactions of *EPDR1* with total fish consumption on CRC risk. Our findings of biological and functional pathways involved in the link between dietary intake and CRC need to be confirmed in future experimental studies.

## Notes

Supplement 1.
eMethods.

eReferences.
**eTable 1.** Dietary Intake and Colorectal Cancer Risk in a Nested Case-Control From the UK Biobank **eTable 2.** Summary of Genetic Variants Suggestively Interacted With Dietary Factors on Colorectal Cancer Risk (p<1x10^-5^) **eTable 3.** Summary of Genetic Variants Mapped to *EPDR1* Gene **eTable 4.** Factor Loading Matrix for Major *EPDR1* Gene Principal Components Identified From SNP Matrix Using Principal Component Analysis **eTable 5.** Associations Between Fish Intake and Colorectal Cancer Risk According to *EPDR1* Gene Pattern Scores **eTable 6.** Dietary Intake Among Study Among Participants Who Completed Touchscreen Questionnaire at Least Two Visit Assessments **eFigure 1.** Manhattan Plot of SNP-Diet Interactions Identified in the Genome-Wide Interaction Analysis of (A) Red Meat, (B) Processed Meat, (C) Poultry, (D) Fish, (E) Milk, and (F) Cheese **eFigure 2.** Manhattan Plot of SNP-Diet Interactions Identified in the Genome-Wide Interaction Analysis of (A) Fruit, (B) Vegetables, (C) Coffee, (D) Tea, and (E) Alcohol **eFigure 3.** Significantly Enriched Gene-Sets Interacted With Milk Consumption **eFigure 4.** Significantly Enriched Gene-Sets Interacted With Cheese Consumption **eFigure 5.** Significantly Enriched Gene-Sets Interacted With Tea Consumption **eFigure 6.** Significantly Enriched Gene-Sets Interacted With Alcohol Consumption **eFigure 7.** Scree Plot for Explained Variance of Principal Components Derived From Multiple Variants Located in *EPDR1* Gene Using Principal Component Analysis **eFigure 8.** Summary of Findings for Gene-Diet Interaction in Colorectal Cancer

Supplement 2.
Data Sharing Statement

## References

[1] Dyba T, Randi G, Bray F, . The European cancer burden in 2020: incidence and mortality estimates for 40 countries and 25 major cancers. Eur J Cancer. 2021;157:308-347. doi:10.1016/j.ejca.2021.07.039 34560371 PMC8568058

[2] Vieira AR, Abar L, Chan DSM, . Foods and beverages and colorectal cancer risk: a systematic review and meta-analysis of cohort studies, an update of the evidence of the WCRF-AICR Continuous Update Project. Ann Oncol. 2017;28(8):1788-1802. doi:10.1093/annonc/mdx171 28407090

[3] Papadimitriou N, Markozannes G, Kanellopoulou A, . An umbrella review of the evidence associating diet and cancer risk at 11 anatomical sites. Nat Commun. 2021;12(1):4579. doi:10.1038/s41467-021-24861-8 34321471 PMC8319326

[4] Hoang T, Cho S, Choi JY, Kang D, Shin A. Genome-wide association study adjusting for familial relatedness identifies novel loci for food intake in the UK Biobank. Research Square Platform LLC. 2023. Accessed January 10, 2024. https://ouci.dntb.gov.ua/en/works/7q3K6y5l/

[5] Kantor ED, Giovannucci EL. Gene-diet interactions and their impact on colorectal cancer risk. Curr Nutr Rep. 2015;4(1):13-21. doi:10.1007/s13668-014-0114-2 25844273 PMC4383391

[6] Doaei S, Hajiesmaeil M, Aminifard A, Mosavi-Jarrahi SA, Akbari ME, Gholamalizadeh M. Effects of gene polymorphisms of metabolic enzymes on the association between red and processed meat consumption and the development of colon cancer; a literature review. J Nutr Sci. 2018;7:e26. doi:10.1017/jns.2018.17 30305892 PMC6176493

[7] Vogtmann E, Xiang YB, Li HL, . Cruciferous vegetables, glutathione *S*-transferase polymorphisms, and the risk of colorectal cancer among Chinese men. Ann Epidemiol. 2014;24(1):44-49. doi:10.1016/j.annepidem.2013.10.003 24238877 PMC3864981

[8] Yang G, Gao YT, Shu XO, . Isothiocyanate exposure, glutathione *S*-transferase polymorphisms, and colorectal cancer risk. Am J Clin Nutr. 2010;91(3):704-711. doi:10.3945/ajcn.2009.28683 20042523 PMC2824157

[9] Kim JK, Shin DH, Park HG, Shin EC. Cruciferous vegetables, glutathione *S*-transferases, and implications of their interaction to colorectal cancer risk: a review. J Korean Soc Appl Biol Chem. 2014;57(4):511-517. doi:10.1007/s13765-014-4014-3

[10] Boyce WT, Sokolowski MB, Robinson GE. Genes and environments, development and time. Proc Natl Acad Sci U S A. 2020;117(38):23235-23241. doi:10.1073/pnas.2016710117 32967067 PMC7519332

[11] Figueiredo JC, Hsu L, Hutter CM, ; CCFR; GECCO. Genome-wide diet-gene interaction analyses for risk of colorectal cancer. PLoS Genet. 2014;10(4):e1004228. doi:10.1371/journal.pgen.1004228 24743840 PMC3990510

[12] Sales NM, Pelegrini PB, Goersch MC. Nutrigenomics: definitions and advances of this new science. J Nutr Metab. 2014;2014:202759. doi:10.1155/2014/202759 24795820 PMC3984860

[13] Farhud D, Yeganeh MZ, Yeganeh MZ. Nutrigenomics and nutrigenetics. Iran J Public Health. 2010;39(4):1-14. 23113033 PMC3481686

[14] Supic G, Zeljic K, Magic Z. Chapter 15: epigenetic nutraceuticals in cancer treatment. In: Holban M, Grumezescu AM, eds. Therapeutic Foods: Handbook of Food Bioengineering. Vol 8. Academic Press; 2018:449-493. doi:10.1016/B978-0-12-811517-6.00015-5

[15] Kohlmeier M. Chapter 5: how does nutrigenetics influence long-term health? In: Nutrigenetics: Applying the Science of Personal Nutrition. Academic Press; 2013.

[16] Song M, Garrett WS, Chan AT. Nutrients, foods, and colorectal cancer prevention. Gastroenterology. 2015;148(6):1244-1260. doi:10.1053/j.gastro.2014.12.035 25575572 PMC4409470

[17] UK Biobank. Accessed January 15, 2024. https://www.ukbiobank.ac.uk/

[18] Canela-Xandri O, Rawlik K, Tenesa A. An atlas of genetic associations in UK Biobank. Nat Genet. 2018;50(11):1593-1599. doi:10.1038/s41588-018-0248-z 30349118 PMC6707814

[19] Bycroft C, Freeman C, Petkova D, . The UK Biobank resource with deep phenotyping and genomic data. Nature. 2018;562(7726):203-209. doi:10.1038/s41586-018-0579-z 30305743 PMC6786975

[20] Sudlow C, Gallacher J, Allen N, . UK biobank: an open access resource for identifying the causes of a wide range of complex diseases of middle and old age. PLoS Med. 2015;12(3):e1001779. doi:10.1371/journal.pmed.1001779 25826379 PMC4380465

[21] Mackenbach JD, Dijkstra SC, Beulens JWJ, . Socioeconomic and ethnic differences in the relation between dietary costs and dietary quality: the HELIUS study. Nutr J. 2019;18(1):21. doi:10.1186/s12937-019-0445-3 30922320 PMC6440156

[22] Wang Y, Chen X. How much of racial/ethnic disparities in dietary intakes, exercise, and weight status can be explained by nutrition- and health-related psychosocial factors and socioeconomic status among US adults? J Am Diet Assoc. 2011;111(12):1904-1911. doi:10.1016/j.jada.2011.09.036 22117667 PMC3225889

[23] Clayton D, Hills M. Statistical Models in Epidemiology. Vol 14. Oxford University Press; 1995:104-105.

[24] Bradbury KE, Young HJ, Guo W, Key TJ. Dietary assessment in UK Biobank: an evaluation of the performance of the touchscreen dietary questionnaire. J Nutr Sci. 2018;7:e6. doi:10.1017/jns.2017.66 29430297 PMC5799609

[25] Marees AT, de Kluiver H, Stringer S, . A tutorial on conducting genome-wide association studies: quality control and statistical analysis. Int J Methods Psychiatr Res. 2018;27(2):e1608. doi:10.1002/mpr.1608 29484742 PMC6001694

[26] Watanabe K, Taskesen E, van Bochoven A, Posthuma D. Functional mapping and annotation of genetic associations with FUMA. Nat Commun. 2017;8(1):1826. doi:10.1038/s41467-017-01261-5 29184056 PMC5705698

[27] Purcell S, Chang C. PLINK 2.00 alpha. Accessed December 12, 2022. https://www.cog-genomics.org/plink/2.0/

[28] R Core Team. R: a language and environment for statistical computing. Version 4.2.2. R Foundation for Statistical Computing. Accessed December 12, 2022. https://www.R-project.org/

[29] International Agency for Research on Cancer. Agents classified by the IARC monographs, volumes 1–135. Accessed September 26, 2022. https://monographs.iarc.who.int/agents-classified-by-the-iarc/

[30] World Cancer Research Fund/American Institute for Cancer Research. Continuous update project expert report 2018: diet, nutrition, physical activity, and colorectal cancer. Accessed September 26, 2022. https://www.wcrf.org/wp-content/uploads/2021/02/Colorectal-cancer-report.pdf

[31] Bradbury KE, Murphy N, Key TJ. Diet and colorectal cancer in UK Biobank: a prospective study. Int J Epidemiol. 2020;49(1):246-258. doi:10.1093/ije/dyz064 30993317 PMC7124508

[32] Zhang P, Lewinger JP, Conti D, Morrison JL, Gauderman WJ. Detecting gene-environment interactions for a quantitative trait in a genome-wide association study. Genet Epidemiol. 2016;40(5):394-403. doi:10.1002/gepi.21977 27230133 PMC5108681

[33] Kooperberg C, Leblanc M. Increasing the power of identifying gene × gene interactions in genome-wide association studies. Genet Epidemiol. 2008;32(3):255-263. doi:10.1002/gepi.20300 18200600 PMC2955421

[34] Murcray CE, Lewinger JP, Gauderman WJ. Gene-environment interaction in genome-wide association studies. Am J Epidemiol. 2009;169(2):219-226. doi:10.1093/aje/kwn353 19022827 PMC2732981

[35] Partlett C, Hall NJ, Leaf A, Juszczak E, Linsell L. Application of the matched nested case-control design to the secondary analysis of trial data. BMC Med Res Methodol. 2020;20(1):117. doi:10.1186/s12874-020-01007-w 32410578 PMC7227268

[36] Bi W, Zhao Z, Dey R, Fritsche LG, Mukherjee B, Lee S. A fast and accurate method for genome-wide scale phenome-wide G × E analysis and its application to UK Biobank. Am J Hum Genet. 2019;105(6):1182-1192. doi:10.1016/j.ajhg.2019.10.008 31735295 PMC6904814

[37] Brown KF, Rumgay H, Dunlop C, . The fraction of cancer attributable to modifiable risk factors in England, Wales, Scotland, Northern Ireland, and the United Kingdom in 2015. Br J Cancer. 2018;118(8):1130-1141. doi:10.1038/s41416-018-0029-6 29567982 PMC5931106

[38] Gong J, Hutter CM, Newcomb PA, ; CCFR and GECCO. Genome-wide interaction analyses between genetic variants and alcohol consumption and smoking for risk of colorectal cancer. PLoS Genet. 2016;12(10):e1006296. doi:10.1371/journal.pgen.1006296 27723779 PMC5065124

[39] Yang T, Li X, Farrington SM, . A systematic analysis of interactions between environmental risk factors and genetic variation in susceptibility to colorectal cancer. Cancer Epidemiol Biomarkers Prev. 2020;29(6):1145-1153. doi:10.1158/1055-9965.EPI-19-1328 32238408 PMC7311198

[40] Caini S, Chioccioli S, Pastore E, . Fish consumption and colorectal cancer risk: meta-analysis of prospective epidemiological studies and review of evidence from animal studies. Cancers (Basel). 2022;14(3):640. doi:10.3390/cancers14030640 35158907 PMC8833371

[41] McDougall C, Hammond MJ, Dailey SC, Somorjai IML, Cummins SF, Degnan BM. The evolution of ependymin-related proteins. BMC Evol Biol. 2018;18(1):182. doi:10.1186/s12862-018-1306-y 30514200 PMC6280359

[42] Yang Y, Zhang H, Liu Z, Zhao F, Liang G. EPDR1 is related to stages and metastasize in bladder cancer and can be used as a prognostic biomarker. BMC Urol. 2021;21(1):71. doi:10.1186/s12894-021-00843-2 33902536 PMC8077848

[43] Gimeno-Valiente F, Riffo-Campos AL, Ayala G, . *EPDR1* up-regulation in human colorectal cancer is related to staging and favours cell proliferation and invasiveness. Sci Rep. 2020;10(1):3723. doi:10.1038/s41598-020-60476-7 32111877 PMC7048834

[44] Chu CH, Chang SC, Wang HH, Yang SH, Lai KC, Lee TC. Prognostic values of *EPDR1* hypermethylation and its inhibitory function on tumor invasion in colorectal cancer. Cancers (Basel). 2018;10(10):393. doi:10.3390/cancers10100393 30360391 PMC6211107

[45] Deshmukh AS, Peijs L, Beaudry JL, . Proteomics-based comparative mapping of the secretomes of human brown and white adipocytes reveals EPDR1 as a novel batokine. Cell Metab. 2019;30(5):963-975. doi:10.1016/j.cmet.2019.10.001 31668873

[46] Yang L, Xiao M, Li X, Tang Y, Wang YL. Arginine ADP-ribosyltransferase 1 promotes angiogenesis in colorectal cancer via the PI3K/Akt pathway. Int J Mol Med. 2016;37(3):734-742. doi:10.3892/ijmm.2016.2473 26847718 PMC4771103

[47] Long WB, Pu X, Tang Y, . Arginine ADP-ribosyltransferase 1 regulates glycolysis in colorectal cancer via the PI3K/AKT/HIF1alpha pathway. Curr Med Sci. 2022;42(4):733-741. doi:10.1007/s11596-022-2606-4 35798928

[48] Miao R, Fang X, Wei J, Wu H, Wang X, Tian J. Akt: a potential drug target for metabolic syndrome. Front Physiol. 2022;13:822333. doi:10.3389/fphys.2022.822333 35330934 PMC8940245

[49] Kim J, Hwang JY, Kim KN, Choi YJ, Chang N, Huh KB. Relationship between milk and calcium intake and lipid metabolism in female patients with type 2 diabetes. Yonsei Med J. 2013;54(3):626-636. doi:10.3349/ymj.2013.54.3.626 23549807 PMC3635625

[50] Trimmer C, Keller A, Murphy NR, . Genetic variation across the human olfactory receptor repertoire alters odor perception. Proc Natl Acad Sci U S A. 2019;116(19):9475-9480. doi:10.1073/pnas.1804106115 31040214 PMC6511007

[51] Rizzo PV, Del Toro-Gipson RS, Cadwallader DC, Drake MA. Identification of aroma-active compounds in cheddar cheese imparted by wood smoke. J Dairy Sci. 2022;105(7):5622-5640. doi:10.3168/jds.2021-21697 35570037

[52] Yoon JY, Kwon HH, Min SU, Thiboutot DM, Suh DH. Epigallocatechin-3-gallate improves acne in humans by modulating intracellular molecular targets and inhibiting *P acnes*. J Invest Dermatol. 2013;133(2):429-440. doi:10.1038/jid.2012.292 23096708

[53] Dai Y, Jiang Z, Qiu Y, Kang Y, Xu H, Xu T. Identification of key carcinogenic genes in colon adenocarcinoma. Iran J Public Health. 2022;51(2):364-374. doi:10.18502/ijph.v51i2.8689 35866125 PMC9273482

[54] Finelli R, Mottola F, Agarwal A. Impact of alcohol consumption on male fertility potential: a narrative review. Int J Environ Res Public Health. 2021;19(1):328. doi:10.3390/ijerph19010328 35010587 PMC8751073

[55] Chen Z, Shi C, Gao S, Song D, Feng Y. Impact of protamine I on colon cancer proliferation, invasion, migration, diagnosis and prognosis. Biol Chem. 2018;399(3):265-275. doi:10.1515/hsz-2017-0222 29140788

[56] Nagirnaja L, Aston KI, Conrad DF. Genetic intersection of male infertility and cancer. Fertil Steril. 2018;109(1):20-26. doi:10.1016/j.fertnstert.2017.10.028 29307395 PMC5761685

[57] de Leeuw CA, Mooij JM, Heskes T, Posthuma D. MAGMA: generalized gene-set analysis of GWAS data. PLoS Comput Biol. 2015;11(4):e1004219. doi:10.1371/journal.pcbi.1004219 25885710 PMC4401657

