# Supplementary material for: Genome-Wide Interaction Study of Dietary Intake and Colorectal Cancer Risk in the UK Biobank
Source: JAMA Netw Open. 2024 Feb 27;7(2):e240465. doi: 10.1001/jamanetworkopen.2024.0465 (PMC10900970; doi:10.1001/jamanetworkopen.2024.0465)
Supplement: Supplement 1. — eMethods. eReferences. eTable 1. Dietary Intake and Colorectal Cancer Risk in a Nested Case-Control From the UK Biobank eTable 2. Summary of Genetic Variants Suggestively Interacted With Dietary Factors on Colorectal Cancer Risk (p<1x10^-5^) eTable 3. Summary of Genetic Variants Mapped to EPDR1 Gene eTable 4. Factor Loading Matrix for Major EPDR1 Gene Principal Components Identified From SNP Matrix Using Principal Component Analysis eTable 5. Associations Between Fish Intake and Colorectal Cancer Risk According to EPDR1 Gene Pattern Scores eTable 6. Dietary Intake Among Study Among Participants Who Completed Touchscreen Questionnaire at Least Two Visit Assessments eFigure 1. Manhattan Plot of SNP-Diet Interactions Identified in the Genome-Wide Interaction Analysis of (A) Red Meat, (B) Processed Meat, (C) Poultry, (D) Fish, (E) Milk, and (F) Cheese eFigure 2. Manhattan Plot of SNP-Diet Interactions Identified in the Genome-Wide Interaction Analysis of (A) Fruit, (B) Vegetables, (C) Coffee, (D) Tea, and (E) Alcohol eFigure 3. Significantly Enriched Gene-Sets Interacted With Milk Consumption eFigure 4. Significantly Enriched Gene-Sets Interacted With Cheese Consumption eFigure 5. Significantly Enriched Gene-Sets Interacted With Tea Consumption eFigure 6. Significantly Enriched Gene-Sets Interacted With Alcohol Consumption eFigure 7. Scree Plot for Explained Variance of Principal Components Derived From Multiple Variants Located in EPDR1 Gene Using Principal Component Analysis eFigure 8. Summary of Findings for Gene-Diet Interaction in Colorectal Cancer [file jamanetwopen-e240465-s001.pdf]

Supplementary Online Content

Hoang T, Cho S, Choi JY, Kang D, Shin A. Genome-wide interaction study of dietary intake and colorectal cancer risk in the UK Biobank. *JAMA Netw Open.* 2024;7(2):e240465. doi:10.1001/jamanetworkopen.2024.0465

eMethods.

eReferences.

eTable 1. Dietary Intake and Colorectal Cancer Risk in a Nested Case-Control from the UK Biobank

eTable 2. Summary of Genetic Variants Suggestively Interacted with Dietary Factors on Colorectal Cancer Risk ($p<1x10^{-5}$)

eTable 3. Summary of Genetic Variants Mapped to *EPDR1* Gene

eTable 4. Factor Loading Matrix for Major *EPDR1* Gene Principal Components Identified from SNP Matrix Using Principal Component Analysis

eTable 5. Associations Between Fish Intake and Colorectal Cancer Risk According to *EPDR1* Gene Pattern Scores

eTable 6. Dietary Intake Among Study Among Participants Who Completed Touchscreen Questionnaire At least Two Visit Assessments

eFigure 1. Manhattan Plot of SNP-Diet Interactions Identified in the Genome-Wide Interaction Analysis of (A) Red Meat, (B) Processed Meat, (C) Poultry, (D) Fish, (E) Milk, and (F) Cheese

eFigure 2. Manhattan Plot of SNP-Diet Interactions Identified in the Genome-Wide Interaction Analysis of (A) Fruit, (B) Vegetables, (C) Coffee, (D) Tea, and (E) Alcohol

eFigure 3. Significantly Enriched Gene-Sets Interacted with Milk Consumption

eFigure 4. Significantly Enriched Gene-Sets Interacted with Cheese Consumption

eFigure 5. Significantly Enriched Gene-Sets Interacted with Tea Consumption

eFigure 6. Significantly Enriched Gene-Sets Interacted with Alcohol Consumption

eFigure 7. Scree Plot for Explained Variance of Principal Components Derived from Multiple Variants Located in *EPDR1* Gene Using Principal Component Analysis

eFigure 8. Summary of Findings for Gene-Diet Interaction in Colorectal Cancer

This supplementary material has been provided by the authors to give readers additional information about their work

© 2024 Hoang T et al. *JAMA Network Open.*

eMethods.

Dietary intake assessment

Information on average dietary intake in the preceding year was obtained via a touchscreen food frequency questionnaire [1]. We combined intake of fresh fruits and dried fruits as total fruits; cooked vegetables and salad/raw vegetables as total vegetables; pork, beef, lamb/mutton as red meat; and oily fish and nonoily fish as total fish [2]. Intake amounts of total fruit (servings/day), vegetables (servings/day), coffee (cups/day), and tea (cups/day) were obtained in continuous form. Intake frequencies of red meat, processed meat, poultry, fish, and cheese were converted to the continuous form (times/week) as follows: 0=never, 0.5=less than once a week, 1=once a week, 3=2-4 times a week, 5.5=5-6 times a week, and 7=once or more daily. Alcohol consumption was converted to the continuous form (times/week) as follows: 0=never, 0.125=special occasions only, 0.5=one to three times a month, 1.5=1-2 times a week, 3.5=3-4 times a week, and 7=daily or almost daily. Milk intake (mL/day) was estimated based on consumption of types of milk, cereals, coffee, and tea [2].

Genotyping, imputation, and quality control

All participants in UK Biobank were genotyped using Affymetrix UK BiLEVE Axiom and Affymetrix Axiom arrays, as described elsewhere [3,4]. Quality control and imputation to UK10K and 1000 Genomes Phase 3 and the Haplotype Reference Consortium reference panel were performed centrally by the Wellcome Trust Centre for Human Genetics and University of Oxford [3]. The data contain a total of 93,095,623 markers. We further applied quality control to ensure inclusion of high-quality variants. SNPs with a low imputation score (imputation score <0.3), high missingness (geno>0.05), low minor allele frequency (maf<0.05), and deviation from the expected Hardy-Weinberg equilibrium ($p<1\times10^{-6}$) were excluded [5], leaving 4,122,345 single-nucleotide polymorphisms (SNPs) for the final genome-wide interaction analysis.

Statistical analysis

Genome-wide interaction analysis was performed for 11 individual dietary phenotypes to identify loci that modify the effects of dietary intake on colorectal cancer (CRC). The SNP-diet

© 2024 Hoang T et al. *JAMA Network Open.*

interaction was captured by testing for the SNP*diet interaction term in the following regression formula:

$$logit(p_i) = \alpha_1 + \sum_{j=2}^{s} \alpha_j z_{ij} + \beta_G G_i + \beta_D D_i + \beta_{GxD} * G_i * D_i + \sum_{k=1}^{8} \beta_{ik} C_{ik} + \epsilon_i$$

where p_i represents the probability of being a CRC case for any individual i, z_{ij} is the binary indicator variable for stratum j among s matching risk sets, G_i is the SNP alternative allele count, D_i is the dietary intake frequency, C_{ik} is the covariates including age, sex, and six principal component scores, and ϵ_i is the residual. α_j is the regression coefficient associated with stratum indicator variables, and β_G, β_D, and β_{GxD} are regression coefficients for SNP, dietary intake, and SNP*diet interaction term, respectively. The p value of the β_{GxD} coefficient was used to assess the existence of an interaction. We determined SNPs that both suggestively (two-sided $p<1x10^{-5}$) and significantly (two-sided $p<5x10^{-8}$) interact with dietary factors on CRC risk. Genotype data were handled in plink2 [6] and imported into the R program (version 4.2.2) [7] for association and interaction analyses.

Apart from genome-wide interaction analysis at the SNP level, gene-based and gene-set enrichment analyses are suggested to exert more powerful results [8]. In gene-based analysis, genetic variants are aggregated to the whole gene level, testing the joint effect of all markers in the gene. In gene-set analysis, individual genes are aggregated into groups of genes sharing certain biological pathways and functions [8]. Given the list of predefined genes, gene-set enrichment analysis searches for sets of genes that are significantly overrepresented [8]. These sets of genes normally consist of genes that function together in a known biological pathway [8].

To investigate whether SNP-diet interaction patterns tended to converge within genetic regions, we carried out a gene-based analysis using MAGMA in web-based FUMA [9]. Overall, input SNPs were mapped to 18,041 protein-coding genes; two-sided $p<2.77\times10^{-6}$ (0.05/18,000) was deemed to be significant in gene-based analysis. Summary statistics from GWI analysis were also used as input files for which each SNP was assigned to a gene using the NCBI 37.3 gene definition. MAGMA further implemented gene-set enrichment analysis to identify Gene Ontology terms and biological pathways from multiple sources [9]. We considered a Benjamini–Hochberg adjusted two-sided p value <0.05 as significant in gene-set enrichment analysis.

To further interpret significant interactions between dietary and genetic factors in CRC risk, we analyzed the association between dietary intake and CRC risk stratified by genetic markers in

© 2024 Hoang T et al. *JAMA Network Open.*

overall 370,004 cancer-free individuals at baseline. Adapting the MAGMA gene-based approach [8], we classified individuals by specific genes (cumulative SNP genotypes) by using the principal component analysis to project SNP matrix into important principal components and performed stratification analyses of diet-CRC associations in subgroups of individuals.

© 2024 Hoang T et al. *JAMA Network Open.*

eReferences.

1. Bradbury KE, Young HJ, Guo W, Key TJ. Dietary assessment in UK Biobank: an evaluation of the performance of the touchscreen dietary questionnaire. *J Nutr Sci*. 2018;7:e6. doi:10.1017/jns.2017.66
2. Bradbury KE, Murphy N, Key TJ. Diet and colorectal cancer in UK Biobank: a prospective study. *Int J Epidemiol*. Feb 1 2020;49(1):246-258. doi:10.1093/ije/dyz064
3. Bycroft C, Freeman C, Petkova D, et al. The UK Biobank resource with deep phenotyping and genomic data. *Nature*. Oct 2018;562(7726):203-209. doi:10.1038/s41586-018-0579-z
4. Sudlow C, Gallacher J, Allen N, et al. UK Biobank: an open access resource for identifying the causes of a wide range of complex diseases of middle and old age. *PLoS Med*. Mar 2015;12(3):e1001779. doi:10.1371/journal.pmed.1001779
5. Marees AT, de Kluiver H, Stringer S, et al. A tutorial on conducting genome-wide association studies: Quality control and statistical analysis. *Int J Methods Psychiatr Res*. Jun 2018;27(2):e1608. doi:10.1002/mpr.1608
6. Purcell S, Chang C. plink2. Accessed December 12, 2022. www.cog-genomics.org/plink/2.0/
7. R Core Team. R: A language and environment for statistical computing. R Foundation for Statistical Computing, Vienna, Austria (version 4.2.2). Accessed December 12, 2022. https://www.R-project.org/
8. de Leeuw CA, Mooij JM, Heskes T, Posthuma D. MAGMA: generalized gene-set analysis of GWAS data. *PLoS Comput Biol*. Apr 2015;11(4):e1004219. doi:10.1371/journal.pcbi.1004219
9. Watanabe K, Taskesen E, van Bochoven A, Posthuma D. Functional mapping and annotation of genetic associations with FUMA. *Nat Commun*. Nov 28 2017;8(1):1826. doi:10.1038/s41467-017-01261-5

© 2024 Hoang T et al. *JAMA Network Open.*

eTable 1. Dietary Intake and Colorectal Cancer Risk in a Nested Case-Control From the UK Biobank

Factor	CRC cases (N=4,686)	Matched controls (N=14,058)	Crude OR (95% CI)	Adjusted OR (95% CI)
**Red meat (times/week)**				
<2	2,086 (44.5)	6,663 (47.4)	1.00 (ref.)	1.00 (ref.)
2 to <3	1,370 (29.2)	4,099 (29.2)	1.07 (0.99-1.16)	1.05 (0.97-1.14)
≥3	1,225 (26.1)	3,280 (23.3)	**1.20 (1.10-1.30)**	**1.16 (1.07-1.27)**
**Processed meat (times/week)**				
<1	1,567 (33.4)	5,107 (36.3)	1.00 (ref.)	1.00 (ref.)
1	1,423 (30.4)	4,225 (30.1)	**1.11 (1.02-1.20)**	1.09 (1.00-1.18)
≥2	1,690 (36.1)	4,701 (33.4)	**1.19 (1.09-1.29)**	**1.16 (1.07-1.26)**
**Poultry (times/week)**				
<1	703 (15.0)	2,275 (16.2)	1.00 (ref.)	1.00 (ref.)
1	1,833 (39.1)	5,410 (38.5)	1.10 (0.99-1.21)	1.08 (0.98-1.20)
≥2	2,142 (45.7)	6,349 (45.2)	1.09 (0.99-1.21)	1.07 (0.96-1.18)
**Total fish (times/week)**				
≤1	1,104 (23.6)	3,308 (23.5)	1.00 (ref.)	1.00 (ref.)
>1 to ≤2	2,272 (48.5)	6,542 (46.5)	1.04 (0.96-1.13)	1.04 (0.96-1.13)
>2	1,303 (27.8)	4,192 (29.8)	0.93 (0.85-1.02)	0.93 (0.85-1.02)
**Milk (100 mL/day)**				
<2	1,640 (35.0)	4,582 (32.6)	1.00 (ref.)	1.00 (ref.)
2 to <3	1,861 (39.7)	5,636 (40.1)	0.92 (0.85-1.00)	0.94 (0.87-1.01)
≥3	989 (21.1)	3,078 (21.9)	**0.90 (0.82-0.98)**	0.92 (0.84-1.01)
**Cheese (times/week)**				
<2	1,849 (39.5)	5,486 (39.1)	1.00 (ref.)	1.00 (ref.)
2 to 4	2,111 (45.0)	6,427 (45.7)	0.98 (0.91-1.05)	0.97 (0.90-1.04)
≥5	609 (13.0)	1,747 (12.4)	1.04 (0.93-1.15)	1.04 (0.94-1.16)
**Total fruit (servings/day)**				
<2	1,644 (35.1)	4,627 (32.9)	1.00 (ref.)	1.00 (ref.)
2 to <4	2,038 (43.5)	6,231 (44.3)	**0.92 (0.85-0.99)**	0.93 (0.86-1.00)
≥4	998 (21.3)	3,176 (22.6)	**0.88 (0.80-0.97)**	**0.90 (0.82-0.99)**
**Total vegetables (servings/day)**				
<4	1,649 (35.2)	4,854 (34.5)	1.00 (ref.)	1.00 (ref.)
<6	1,614 (34.4)	4,879 (34.7)	0.97 (0.90-1.05)	0.97 (0.90-1.05)
≥6	1,384 (29.5)	4,241 (30.2)	0.96 (0.88-1.04)	0.95 (0.87-1.03)
**Coffee (cups/day)**				
<1	1,258 (26.8)	3,730 (26.5)	1.00 (ref.)	1.00 (ref.)
1 to ≤2	1,805 (38.5)	5,701 (40.6)	0.94 (0.86-1.02)	0.93 (0.86-1.01)

© 2024 Hoang T et al. *JAMA Network Open.*

Factor	CRC cases (N=4,686)	Matched controls (N=14,058)	Crude OR (95% CI)	Adjusted OR (95% CI)
>2	1,614 (34.4)	4,605 (32.8)	1.04 (0.95-1.13)	1.01 (0.93-1.10)
**Tea (cups/day)**				
<3	1,898 (40.5)	5,447 (38.7)	1.00 (ref.)	1.00 (ref.)
<5	1,413 (30.2)	4,254 (30.3)	0.95 (0.88-1.03)	0.97 (0.89-1.05)
≥5	1,367 (29.2)	4,321 (30.7)	**0.91 (0.84-0.98)**	0.93 (0.85-1.00)
**Alcohol (times/week)**				
<1	1,212 (25.9)	3,820 (27.2)	1.00 (ref.)	1.00 (ref.)
1 to 2	1,127 (24.1)	3,514 (25.0)	1.01 (0.92-1.11)	1.02 (0.93-1.12)
≥3	2,340 (49.9)	6,713 (47.8)	**1.10 (1.02-1.20)**	**1.10 (1.01-1.20)**

CRC, colorectal cancer; OR, odds ratio; CI, confidence interval. The ORs were estimated using conditional logistic regression models with adjustment for smoking (never, former, and current smokers), drinking (never or rarely, once a month to twice a week, 3-4 times/day, and daily or more, except for exposure of alcohol), body mass index (BMI) (<25, 25 to <30, and ≥30 kg/m2), and physical activity (met and unmet the 2017 UK physical activity guidelines of 150 minutes of walking or moderate activity per week or 75 minutes of vigorous activity). Bold font indicates significant difference.

© 2024 Hoang T et al. *JAMA Network Open.*

eTable 2. Summary of Genetic Variants Suggestively Interacted With Dietary Factors on Colorectal Cancer Risk ($p<1x10^{-5}$)

Chromosome	Variant	Position	Alternative allele	Reference allele	Beta	SE	P-value
**Red meat**							
2	rs10180334	58917026	A	G	-0.242	0.0540	7.54e-06
2	rs6729841	148346874	A	G	0.170	0.0379	7.53e-06
2	rs2118769	148347811	C	T	0.170	0.0379	7.53e-06
2	rs13428299	216523164	G	A	-0.281	0.0624	6.61e-06
2	rs13428305	216523167	G	A	-0.282	0.0625	6.46e-06
3	3:1443811	1443811	C	CA	-0.198	0.0440	6.90e-06
3	rs116215027	117135170	T	C	0.406	0.0837	1.27e-06
4	rs2546249	181596898	C	T	-0.167	0.0371	6.80e-06
4	rs2613072	181598141	G	A	-0.168	0.0374	6.58e-06
4	rs2546248	181598667	A	T	-0.184	0.0380	1.28e-06
7	rs35173185	154435563	C	G	-0.192	0.0377	3.40e-07
11	rs4930640	68800370	G	A	0.184	0.0404	5.46e-06
20	rs199562	19941538	T	A	0.199	0.0442	6.37e-06
**Processed meat**							
1	rs2722780	5255304	T	G	0.193	0.0432	8.11e-06
1	rs2722781	5257229	T	C	0.201	0.0435	3.82e-06
1	rs2679217	5258982	C	A	0.202	0.0435	3.33e-06
1	rs2722788	5265438	T	C	0.187	0.0395	2.23e-06
11	rs1425878	127971680	A	G	-0.167	0.0379	9.98e-06
11	rs4995093	127984138	A	G	-0.172	0.0378	5.13e-06
11	rs713069	127986917	T	C	-0.171	0.0377	6.22e-06
11	rs10893820	127987031	A	G	-0.171	0.0377	6.22e-06
16	rs56299822	54007722	G	A	0.253	0.0571	9.46e-06
16	rs7192835	54008455	T	C	0.253	0.0568	8.55e-06
16	rs9302654	54009545	T	C	0.215	0.0461	3.10e-06
16	rs13337356	54017310	G	C	0.216	0.0458	2.39e-06
**Poultry**							
2	rs1404774	216139373	A	C	-0.279	0.0565	8.12e-07
4	rs2131004	173015627	A	T	-0.221	0.0457	1.30e-06
4	rs2172194	173015965	A	C	-0.222	0.0456	1.10e-06
7	rs7808932	51811086	A	G	0.460	0.0968	1.97e-06
14	rs17808556	62599447	G	A	0.384	0.0858	7.81e-06
14	rs77182825	100011046	T	C	0.437	0.0966	6.11e-06
**Total fish**							
3	rs62270506	125494043	A	G	-0.261	0.0577	6.11e-06
3	rs61790516	170554919	G	A	-0.429	0.0924	3.51e-06

© 2024 Hoang T et al. *JAMA Network Open.*

Chromosome	Variant	Position	Alternative allele	Reference allele	Beta	SE	P-value
3	rs61790536	170574562	C	G	-0.425	0.0923	4.13e-06
3	rs61790537	170574598	G	A	-0.425	0.0923	4.13e-06
3	rs61790538	170576880	T	C	-0.422	0.0925	4.94e-06
4	rs2718446	148549000	C	T	-0.377	0.0841	7.46e-06
4	rs72953456	148658597	T	C	-0.370	0.0834	9.33e-06
4	rs12642040	156720135	A	G	-0.240	0.0511	2.78e-06
4	rs2229202	156721198	T	C	-0.243	0.0512	2.09e-06
6	rs9354216	65996855	T	C	-0.215	0.0442	1.11e-06
7	rs2122624	37867052	G	A	-0.153	0.0342	7.48e-06
7	rs12539845	137952273	A	C	0.167	0.0359	3.17e-06
7	rs12534619	137952277	C	A	0.161	0.0353	5.02e-06
10	rs56407831	82010606	C	T	-0.178	0.0376	2.25e-06
12	rs78636548	41905790	A	G	0.274	0.0618	9.45e-06
12	rs2730983	43127101	T	C	-0.146	0.0330	9.80e-06
12	rs1420413	98199400	C	T	-0.157	0.0341	4.52e-06
12	rs10860243	98212294	G	A	-0.172	0.0342	4.90e-07
12	rs7310376	98212884	G	A	-0.178	0.0345	2.60e-07
12	rs7310984	98213212	T	C	-0.182	0.0346	1.39e-07
12	rs823559	101530109	G	A	0.320	0.0691	3.57e-06
12	rs10744738	119787246	G	A	-0.153	0.0340	6.37e-06
12	rs4238062	119789218	T	C	-0.155	0.0340	4.98e-06
12	rs5801334	119791883	CTT	C	-0.153	0.0346	9.47e-06
12	rs12369461	119798476	G	A	-0.154	0.0343	7.32e-06
12	rs7975869	119798542	G	A	-0.154	0.0343	7.63e-06
12	rs10774494	119813129	G	A	-0.153	0.0340	6.81e-06
12	rs7313792	119823625	T	C	-0.145	0.0327	9.94e-06
18	rs1791072	3353652	T	C	0.198	0.0447	9.44e-06
18	rs16971133	36492686	G	A	-0.229	0.0506	6.34e-06
18	rs112155595	36498378	T	C	-0.226	0.0506	8.31e-06
18	rs112356584	36499506	T	A	-0.228	0.0506	6.71e-06
18	rs57697414	36501628	A	G	-0.226	0.0507	7.98e-06
19	rs4807543	1106107	T	G	0.377	0.0734	2.75e-07
**Milk**							
1	rs11260752	16742820	C	T	-0.261	0.0556	2.67e-06
1	rs12145688	16743342	G	T	-0.261	0.0555	2.54e-06
1	rs12145711	16743398	C	T	-0.260	0.0555	2.74e-06
3	rs939054	32903723	A	C	-0.251	0.0552	5.29e-06
4	rs17073249	183328586	A	C	-0.488	0.1083	6.45e-06
4	rs17073255	183330031	T	C	-0.494	0.1090	5.70e-06
4	rs17073259	183330801	C	T	-0.494	0.1090	5.70e-06
4	rs72699967	183331179	T	C	-0.494	0.1088	5.53e-06

© 2024 Hoang T et al. *JAMA Network Open.*

Chromosome	Variant	Position	Alternative allele	Reference allele	Beta	SE	P-value
4	rs17073264	183331890	A	G	-0.494	0.1090	5.70e-06
4	rs72699969	183332107	T	C	-0.493	0.1088	6.00e-06
4	rs17073266	183333090	T	G	-0.489	0.1087	6.93e-06
4	rs72699971	183334497	T	C	-0.495	0.1090	5.53e-06
4	rs10520531	183335191	T	C	-0.494	0.1090	5.77e-06
4	rs6849034	183335675	T	C	-0.489	0.1088	7.02e-06
4	rs72699979	183338562	G	A	-0.483	0.1081	7.99e-06
4	rs72699980	183338568	A	G	-0.483	0.1081	7.99e-06
4	rs72699998	183355479	C	A	-0.472	0.1045	6.38e-06
4	rs72699999	183355532	T	C	-0.472	0.1045	6.38e-06
4	rs777981116	183366764	AT	A	-0.505	0.1085	3.26e-06
4	rs12505033	183367131	C	T	-0.472	0.1059	8.35e-06
6	rs17292811	90333599	G	A	0.339	0.0757	7.55e-06
6	6:90581653	90581653	C	CT	-0.474	0.1023	3.63e-06
7	rs6959050	12128392	A	G	0.258	0.0579	8.61e-06
7	rs6958807	12128426	T	C	0.257	0.0580	9.46e-06
7	rs59161415	12130536	A	G	0.280	0.0587	1.91e-06
11	rs28867119	3673277	C	A	0.320	0.0708	6.34e-06
19	rs73006731	19810628	C	T	0.256	0.0563	5.44e-06
19	rs11666446	19813345	G	A	0.254	0.0563	6.38e-06
19	rs112373548	19818943	TTTTA	T	0.270	0.0595	5.84e-06
19	rs56129543	19820475	C	G	0.261	0.0562	3.52e-06
19	rs12973486	51069285	A	T	-0.323	0.0721	7.31e-06
**Cheese**							
5	rs148986595	2521881	T	G	-0.284	0.0627	6.08e-06
5	rs2399716	5184128	T	C	-0.217	0.0477	5.59e-06
5	rs7705368	5193888	G	A	-0.197	0.0440	7.93e-06
5	rs2081852	5196007	A	G	-0.195	0.0438	8.57e-06
6	rs332583	48012271	G	T	0.141	0.0317	9.07e-06
9	rs6477925	115155264	C	T	-0.140	0.0308	5.86e-06
9	rs6477926	115155302	C	T	-0.140	0.0308	5.86e-06
9	rs6477928	115156039	G	C	-0.140	0.0309	6.03e-06
9	rs72757495	115159693	C	T	-0.140	0.0308	5.35e-06
9	rs58515578	115160215	G	A	-0.140	0.0308	5.28e-06
9	rs35938444	115160305	C	G	-0.140	0.0309	5.55e-06
9	rs11496554	115160920	A	T	-0.140	0.0308	5.28e-06
9	rs7032244	115161481	C	T	-0.140	0.0308	5.28e-06
9	rs7019825	115161994	C	G	-0.140	0.0308	5.73e-06
9	rs4978488	115163067	G	A	-0.139	0.0309	6.46e-06
9	rs34600812	115164830	TAA	T	-0.137	0.0310	9.38e-06
11	rs17126120	121981729	A	T	0.241	0.0503	1.68e-06

© 2024 Hoang T et al. *JAMA Network Open.*

Chromosome	Variant	Position	Alternative allele	Reference allele	Beta	SE	P-value
11	rs602046	121983986	A	G	-0.229	0.0500	4.43e-06
11	rs482854	121984236	C	T	-0.229	0.0500	4.43e-06
11	rs17126145	121985355	A	T	0.228	0.0499	4.81e-06
11	rs61903717	121985467	G	C	0.228	0.0499	4.81e-06
11	rs17126153	121985725	G	A	0.228	0.0499	4.83e-06
11	rs7103734	121991136	C	G	0.226	0.0498	5.58e-06
11	rs17246402	121993571	A	G	0.226	0.0498	5.58e-06
11	rs7946064	121995706	T	G	0.226	0.0498	5.58e-06
11	rs12794777	121996191	G	C	0.226	0.0498	5.66e-06
11	rs17126165	121997282	T	C	0.234	0.0497	2.49e-06
11	rs36046125	121998212	T	C	0.229	0.0498	4.31e-06
11	rs35529859	121998323	C	T	0.237	0.0496	1.76e-06
11	rs4935788	122004498	T	A	0.228	0.0496	4.43e-06
11	rs4936672	122005286	C	A	0.238	0.0496	1.57e-06
11	rs7936494	122005632	C	G	0.238	0.0496	1.58e-06
11	rs4936673	122009051	A	G	0.229	0.0497	3.82e-06
11	rs12420771	122011734	A	T	0.234	0.0496	2.48e-06
11	rs67806370	122013474	G	A	0.234	0.0496	2.43e-06
11	rs12421791	122019261	C	T	0.234	0.0496	2.32e-06
11	rs4936674	122021283	A	C	0.237	0.0502	2.27e-06
11	rs1364757	122021460	A	G	0.237	0.0502	2.35e-06
11	rs140603317	123899756	GC	G	0.248	0.0544	5.31e-06
12	rs7974391	89513761	C	T	0.174	0.0391	8.64e-06
12	rs1983374	89525052	A	G	0.187	0.0389	1.54e-06
12	rs10777153	89531829	C	G	0.192	0.0390	9.16e-07
12	rs10777154	89543164	A	G	0.192	0.0388	6.99e-07
12	rs7969537	89543809	T	A	0.193	0.0389	7.31e-07
12	rs7133167	89547580	T	A	0.185	0.0387	1.70e-06
12	rs1915028	89553002	C	T	0.186	0.0387	1.61e-06
12	rs7974399	89568297	T	C	0.186	0.0388	1.68e-06
12	rs1381859	89578982	A	T	0.183	0.0389	2.43e-06
12	rs995727	89589948	A	G	0.182	0.0388	2.74e-06
12	rs151337361	89601404	ATATAT GTGTGT GTGTT	A	0.183	0.0388	2.46e-06
12	rs4842484	89602741	A	C	0.184	0.0388	2.09e-06
12	rs10777157	89603919	C	T	0.183	0.0388	2.28e-06
12	rs899003	89609990	T	C	0.182	0.0387	2.58e-06
15	rs78765970	100382324	C	T	0.206	0.0454	5.60e-06
16	rs3102336	89442006	T	C	-0.137	0.0309	8.79e-06
16	rs3102337	89442034	G	A	-0.137	0.0306	8.24e-06
16	rs3102338	89442250	G	C	-0.138	0.0309	8.55e-06

© 2024 Hoang T et al. *JAMA Network Open.*

Chromosome	Variant	Position	Alternative allele	Reference allele	Beta	SE	P-value
16	rs2965822	89444677	T	A	-0.140	0.0307	5.26e-06
**Total fruit**							
1	rs1417584	231819050	C	T	0.119	0.0268	9.49e-06
1	rs764943985	233964653	T	TTGTTGA	-0.268	0.0601	8.08e-06
1	rs17641257	239300943	G	A	-0.187	0.0417	6.89e-06
2	rs12475352	77588425	T	A	-0.120	0.0272	9.32e-06
2	rs12475354	77588441	C	A	-0.120	0.0272	9.44e-06
4	rs28379432	3808774	C	T	0.239	0.0512	2.89e-06
4	rs28371008	3815134	T	C	0.201	0.0445	6.28e-06
5	rs2561454	66382156	G	A	0.151	0.0338	7.91e-06
5	rs58733230	80300091	T	A	0.172	0.0361	2.01e-06
5	rs9293828	80312586	C	T	0.164	0.0345	1.83e-06
5	rs6874973	80313771	A	C	0.165	0.0346	1.83e-06
5	rs57037802	82276628	T	G	0.172	0.0365	2.62e-06
5	rs62371773	82278274	T	C	0.174	0.0371	2.75e-06
5	rs73364836	166257308	C	T	0.172	0.0378	5.52e-06
6	rs611948	137552949	C	T	-0.154	0.0346	8.50e-06
6	rs1418825	137595896	G	A	-0.155	0.0350	9.66e-06
6	rs2797665	137599914	G	T	-0.156	0.0352	8.92e-06
6	rs1935888	137600146	A	C	-0.157	0.0352	8.04e-06
8	rs4458863	79291252	C	T	-0.144	0.0324	8.35e-06
8	rs12679066	79300759	T	C	-0.144	0.0324	8.35e-06
8	rs13253475	79304170	A	G	-0.146	0.0324	7.06e-06
8	rs36036472	79320466	C	T	-0.147	0.0325	6.06e-06
8	rs13250673	79324892	A	G	-0.148	0.0325	5.40e-06
8	rs10957890	79332176	A	G	-0.150	0.0324	3.71e-06
8	rs6981105	79333400	G	A	-0.151	0.0323	2.99e-06
9	rs11145764	139400406	T	C	0.128	0.0272	2.53e-06
9	rs4880098	139400904	T	C	0.126	0.0272	3.35e-06
9	rs3829116	139401577	G	C	0.128	0.0274	3.19e-06
11	rs71463681	70386479	GC	G	-0.140	0.0286	9.41e-07
11	rs4980608	70387122	G	A	-0.134	0.0282	2.11e-06
12	rs61930461	113821098	T	C	-0.342	0.0731	2.88e-06
12	rs116922807	113822289	G	A	-0.319	0.0689	3.51e-06
12	rs76401723	113838971	C	G	-0.335	0.0699	1.69e-06
12	rs1872716	129568010	A	G	-0.167	0.0334	6.05e-07
12	rs966585	129583901	C	T	-0.179	0.0338	1.25e-07
14	rs2297617	20811588	C	T	-0.136	0.0302	6.33e-06
17	rs7405808	71967060	C	A	-0.127	0.0280	6.32e-06
17	17:71970801	71970801	T	TA	-0.121	0.0267	5.49e-06
20	rs1923101	2303667	G	A	-0.144	0.0315	4.86e-06

© 2024 Hoang T et al. *JAMA Network Open.*

Chromosome	Variant	Position	Alternative allele	Reference allele	Beta	SE	P-value
20	rs2014045	2304832	C	G	-0.138	0.0310	8.22e-06
**Coffee**							
1	rs1332957	170274848	G	A	-0.315	0.0640	8.34e-07
2	rs60091455	75563980	C	G	0.204	0.0457	8.50e-06
6	rs72839123	17897105	A	G	0.229	0.0461	7.16e-07
6	rs72839144	18011452	T	C	0.213	0.0476	7.50e-06
6	rs76038912	121110659	C	T	0.255	0.0574	9.04e-06
7	rs34294791	30364111	A	G	-0.229	0.0483	2.03e-06
7	rs2108794	156447708	A	G	0.184	0.0397	3.58e-06
8	rs9644279	3582903	C	G	0.186	0.0412	6.60e-06
8	rs7824086	139990834	T	G	0.251	0.0563	8.42e-06
12	rs6487450	25232120	A	T	-0.245	0.0513	1.75e-06
12	12:25232997	25232997	T	TG	-0.236	0.0513	4.19e-06
12	rs1683165	25233247	T	A	-0.236	0.0515	4.39e-06
12	rs10743537	25236543	A	C	0.203	0.0435	3.05e-06
12	rs11106954	93695298	C	T	-0.190	0.0422	6.30e-06
12	rs11106955	93695441	T	C	-0.190	0.0422	6.30e-06
12	rs12318123	93695694	C	T	-0.190	0.0422	6.30e-06
12	rs10859478	93696189	G	C	-0.190	0.0422	6.30e-06
12	rs11106958	93697472	C	T	-0.191	0.0422	5.99e-06
12	rs61942901	132242145	C	G	-0.283	0.0642	9.98e-06
12	rs61942902	132243321	G	T	-0.284	0.0642	9.41e-06
12	rs61942903	132246093	T	G	-0.285	0.0639	8.19e-06
12	rs61942904	132246747	T	C	-0.285	0.0639	8.04e-06
12	rs56020999	132253225	T	C	-0.283	0.0636	8.59e-06
12	rs55645619	132254265	A	G	-0.283	0.0636	8.59e-06
12	rs61942910	132254780	A	G	-0.283	0.0636	8.59e-06
12	rs61942911	132257164	A	G	-0.282	0.0636	9.11e-06
12	rs61942912	132257356	A	G	-0.282	0.0636	9.11e-06
12	rs61942913	132257854	C	G	-0.282	0.0636	9.06e-06
12	rs61942914	132258749	T	G	-0.282	0.0636	9.11e-06
12	rs61942915	132261101	A	G	-0.285	0.0637	7.94e-06
12	rs61943860	132267561	T	A	-0.285	0.0637	7.94e-06
12	12:132269780	132269780	A	AACTG	-0.283	0.0637	8.76e-06
12	rs55690718	132276324	A	T	-0.286	0.0638	7.60e-06
12	rs61943864	132278493	C	G	-0.287	0.0638	6.89e-06
12	rs61943865	132278566	G	T	-0.287	0.0638	6.89e-06
12	rs56008725	132282861	G	A	-0.287	0.0638	6.78e-06
12	rs61943867	132282951	A	G	-0.287	0.0638	6.76e-06
12	rs77439309	132283801	C	G	-0.287	0.0637	6.77e-06
12	rs61943868	132285983	A	G	-0.303	0.0664	4.85e-06

© 2024 Hoang T et al. *JAMA Network Open.*

Chromosome	Variant	Position	Alternative allele	Reference allele	Beta	SE	P-value
12	rs61943871	132286299	G	C	-0.288	0.0640	6.97e-06
12	rs61943872	132286453	A	G	-0.290	0.0641	6.17e-06
12	rs61943873	132287202	T	C	-0.288	0.0640	6.63e-06
13	rs17063135	42837205	A	T	0.184	0.0409	6.49e-06
13	rs770397	51149703	T	G	0.337	0.0738	4.83e-06
15	rs2654529	97283092	T	G	0.129	0.0289	8.20e-06
18	rs11387001	72771757	TA	T	-0.178	0.0363	9.69e-07
20	rs8123351	2002100	T	A	-0.313	0.0658	1.93e-06
21	rs8128869	20362274	G	A	0.171	0.0368	3.46e-06
21	rs8129319	20362621	G	A	0.173	0.0363	2.03e-06
21	rs8126544	20365466	C	A	0.169	0.0363	3.26e-06
21	rs76576862	20366447	A	G	0.169	0.0363	3.17e-06
21	rs2825260	20369342	C	T	0.169	0.0362	3.23e-06
21	rs12483354	20371580	T	C	0.163	0.0359	5.90e-06
21	rs2825262	20371878	G	A	0.167	0.0359	3.34e-06
21	rs2825264	20372569	G	T	0.158	0.0341	3.77e-06
21	rs6517849	20374020	T	C	0.160	0.0341	2.54e-06
21	rs77909963	20374574	T	C	0.168	0.0357	2.55e-06
21	rs75961541	20377600	C	T	0.171	0.0357	1.68e-06
21	rs142219204	20378360	GAGT	G	-0.164	0.0341	1.59e-06
21	rs375077125	20381759	AAT	A	0.181	0.0367	8.44e-07
21	rs77677812	20384311	A	C	0.171	0.0354	1.33e-06
21	rs75701928	20384677	T	C	0.172	0.0354	1.26e-06
21	rs9754187	20386608	C	T	0.175	0.0349	5.38e-07
21	rs74709572	20386852	A	G	0.175	0.0350	5.50e-07
21	rs949585	20387556	A	G	0.175	0.0350	5.58e-07
21	rs78000374	20388906	GT	G	0.174	0.0350	7.06e-07
21	rs9789906	20389440	T	C	0.177	0.0353	5.41e-07
21	rs2825275	20393716	T	A	0.168	0.0350	1.64e-06
21	rs76878614	20394213	G	A	0.179	0.0349	2.71e-07
21	rs422059	20394460	G	A	-0.180	0.0349	2.75e-07
21	rs373378	20394678	T	A	-0.181	0.0349	2.18e-07
21	rs387851	20395519	T	C	-0.172	0.0348	7.92e-07
21	rs389467	20395964	A	C	-0.180	0.0350	2.79e-07
21	rs2825278	20397505	C	A	0.181	0.0349	2.29e-07
21	rs2825279	20397995	G	C	0.180	0.0349	2.62e-07
21	rs113773990	20398339	T	G	0.177	0.0348	3.64e-07
21	21:48065015	48065015	T	TA	0.130	0.0275	2.16e-06
22	rs768992480	43440761	C	CAG	0.193	0.0435	9.10e-06
22	rs9608000	43472496	A	G	0.197	0.0441	8.02e-06
22	rs9611998	43472514	C	T	0.198	0.0441	6.91e-06

© 2024 Hoang T et al. *JAMA Network Open.*

Chromosome	Variant	Position	Alternative allele	Reference allele	Beta	SE	P-value
**Tea**							
4	rs28731389	132295955	C	T	-0.090	0.0204	9.90e-06
4	rs12644626	132299611	C	T	-0.092	0.0205	7.94e-06
4	rs4863842	132318919	A	G	-0.091	0.0203	7.45e-06
4	rs4863843	132318973	C	T	-0.091	0.0203	7.45e-06
4	rs11942004	132322797	T	C	-0.091	0.0203	7.45e-06
4	rs12640544	132333485	A	G	-0.092	0.0203	5.82e-06
5	rs39958	55568856	A	G	-0.104	0.0230	6.16e-06
5	rs1864944	147183801	G	A	-0.096	0.0217	9.08e-06
6	6:30436369	30436369	C	CAT	0.115	0.0253	5.82e-06
7	rs550448	28229042	A	G	0.136	0.0308	9.78e-06
9	rs7859864	1777803	C	T	-0.094	0.0212	9.69e-06
9	rs7028588	1786335	T	C	-0.096	0.0213	7.28e-06
12	rs17733831	53029844	C	G	0.101	0.0216	2.79e-06
12	rs12423256	53033600	A	G	0.098	0.0215	5.13e-06
12	rs12811440	53034403	G	T	0.098	0.0216	5.74e-06
13	rs9517132	98575843	T	C	0.101	0.0222	5.44e-06
14	rs17447314	31394538	C	A	0.211	0.0470	7.51e-06
**Alcohol**							
2	rs62188900	150681088	T	C	-0.194	0.0402	1.32e-06
2	rs72890074	185279829	T	G	-0.113	0.0251	6.18e-06
5	rs6861592	160633163	C	A	0.097	0.0195	5.95e-07
6	rs75679567	103756924	T	C	-0.161	0.0364	9.24e-06
11	rs71482039	29734467	A	C	-0.156	0.0345	6.10e-06
11	rs10835571	29805783	T	C	-0.155	0.0344	6.86e-06
11	rs12363095	29805995	A	G	-0.155	0.0344	6.86e-06
11	rs7124294	29806730	G	A	-0.155	0.0344	6.86e-06
11	rs12270721	29815133	G	T	-0.156	0.0344	6.18e-06
11	rs10160512	29816171	A	C	-0.16	0.0346	3.85e-06
11	rs10160514	29816208	A	G	-0.156	0.0344	6.19e-06
11	rs10835584	29817006	A	G	-0.155	0.0344	6.47e-06
11	rs141300095	29817574	G	A	-0.157	0.0346	5.54e-06
11	rs11030823	29817952	A	G	-0.13	0.0293	9.77e-06
11	rs11030824	29819494	A	C	-0.156	0.0345	6.26e-06
11	rs10835587	29819704	C	G	-0.156	0.0345	6.58e-06
11	rs11030826	29820513	T	C	-0.152	0.0343	9.20e-06
11	rs78215038	44064494	T	A	0.115	0.0257	7.60e-06
12	rs35430548	17573701	AT	A	-0.092	0.0203	6.12e-06
12	rs10506075	31682988	C	T	-0.158	0.0333	2.31e-06
12	rs151064790	31754155	G	C	-0.157	0.0345	5.22e-06
12	rs766100385	31755300	A	AC	-0.155	0.0346	7.84e-06

© 2024 Hoang T et al. *JAMA Network Open.*

Chromosome	Variant	Position	Alternative allele	Reference allele	Beta	SE	P-value
16	rs72773814	11401479	T	C	0.127	0.0286	9.11e-06
16	rs3972708	11415633	G	A	0.132	0.0285	3.82e-06
16	rs72773837	11417682	C	T	0.135	0.0285	2.34e-06
16	rs76886907	11421427	T	C	0.13	0.0285	4.83e-06
16	rs56944133	11436669	T	C	0.13	0.0281	3.85e-06
16	rs72773848	11438701	G	C	0.131	0.0284	4.07e-06
16	rs753895396	11442700	G	GCT	0.132	0.0287	4.34e-06
16	rs72773852	11444028	T	C	0.132	0.0285	3.47e-06
16	rs72773859	11447233	T	C	0.131	0.0285	4.08e-06
16	rs72773860	11447920	T	C	0.134	0.0289	3.77e-06
20	rs73096872	32458332	C	G	0.134	0.0296	5.79e-06

© 2024 Hoang T et al. *JAMA Network Open.*

eTable 3. Summary of Genetic Variants Mapped to *EPDR1* Gene

Chr	Variant	Position	Alternative allele	Reference allele	MAF	Beta	SE	P-value
7	rs6945437	37788131	A	G	0.351	-0.127	0.0360	4.24e-04
7	rs10243140	37789556	A	G	0.351	-0.131	0.0360	2.72e-04
7	rs62463305	37790299	T	G	0.351	-0.125	0.0360	5.42e-04
7	rs28407053	37790682	A	G	0.442	-0.132	0.0339	1.02e-04
7	rs10250481	37790924	G	C	0.441	-0.133	0.0338	8.63e-05
7	rs62463306	37793621	A	G	0.356	-0.136	0.0356	1.31e-04
7	rs6954646	37802524	G	A	0.357	-0.138	0.0354	9.43e-05
7	rs10278735	37803161	T	C	0.316	-0.142	0.0363	8.80e-05
7	rs4723706	37808132	C	G	0.370	-0.117	0.0346	7.39e-04
7	rs4142516	37811025	A	G	0.370	-0.112	0.0346	1.16e-03
7	rs760694845	37815538	A	AC	0.348	-0.145	0.0357	5.06e-05
7	rs2392520	37819960	A	C	0.348	-0.146	0.0355	4.07e-05
7	rs2392519	37819961	T	A	0.346	-0.152	0.0357	2.06e-05
7	rs73121244	37827426	T	G	0.348	-0.145	0.0355	4.61e-05
7	rs4720260	37827779	C	G	0.348	-0.144	0.0355	4.79e-05
7	rs10242196	37829792	T	C	0.359	-0.135	0.0351	1.18e-04
7	rs10245272	37829952	T	C	0.359	-0.134	0.0351	1.35e-04
7	rs10249901	37831294	T	C	0.359	-0.134	0.0351	1.33e-04
7	rs10257984	37833185	T	C	0.366	-0.129	0.0348	2.19e-04
7	rs17825495	37833628	G	A	0.359	-0.133	0.0350	1.53e-04
7	rs147171986	37833789	TTA	T	0.357	-0.137	0.0352	9.55e-05
7	7:37834268	37834268	C	CA	0.348	-0.146	0.0356	4.32e-05
7	rs780986521	37836971	A	AGG	0.355	-0.139	0.0353	8.13e-05
7	rs12539577	37837335	A	G	0.355	-0.134	0.0351	1.36e-04
7	7:37837731	37837731	T	TCCTCTCCCA	0.355	-0.137	0.0352	9.59e-05
7	rs2709110	37837826	T	C	0.376	0.132	0.0346	1.40e-04
7	rs2718059	37842065	C	A	0.376	0.133	0.0346	1.21e-04
7	rs10234857	37843650	G	T	0.348	-0.143	0.0354	5.11e-05
7	rs62464362	37845652	C	A	0.348	-0.143	0.0354	5.26e-05
7	rs201375814	37847247	G	A	0.345	-0.136	0.0358	1.40e-04
7	rs779355157	37848514	A	AC	0.349	-0.142	0.0354	6.31e-05
7	7:37849687	37849687	T	TA	0.392	-0.137	0.0356	1.19e-04
7	7:37850005	37850005	G	GA	0.348	-0.139	0.0355	9.45e-05
7	rs1986714	37850129	G	A	0.348	-0.143	0.0354	5.61e-05
7	rs28867163	37850799	G	A	0.349	-0.140	0.0354	7.63e-05
7	rs28858459	37850806	A	G	0.349	-0.140	0.0354	7.63e-05
7	rs10241576	37859423	T	C	0.349	-0.142	0.0354	6.20e-05
7	rs1450847	37860475	C	T	0.378	0.127	0.0345	2.23e-04
7	rs35202238	37861358	GA	G	0.371	0.139	0.0350	6.68e-05

© 2024 Hoang T et al. *JAMA Network Open.*

Chr	Variant	Position	Alternative allele	Reference allele	MAF	Beta	SE	P-value
7	rs1597550	37863043	G	A	0.369	0.132	0.0347	1.39e-04
7	rs2392516	37863185	A	G	0.349	-0.141	0.0354	6.72e-05
7	rs62459519	37864635	G	A	0.349	-0.141	0.0354	6.66e-05
7	rs1376256	37864947	G	A	0.376	0.128	0.0345	1.97e-04
7	rs13437867	37865196	G	A	0.349	-0.141	0.0354	6.66e-05
7	rs2122624	37867052	G	A	0.422	-0.153	0.0342	7.48e-06
7	rs1530819	37867759	A	G	0.350	-0.145	0.0355	4.49e-05

Chr, chromosome; MAF, minor allele frequency; SE, standard error.

© 2024 Hoang T et al. *JAMA Network Open.*

eTable 4. Factor Loading Matrix for Major *EPDR1* Gene Principal Components Identified From SNP Matrix Using Principal Component Analysis

Variant	Factor 1	Factor 2	Factor 3
rs6945437	-0.15	-0.1	0.04
rs10243140	-0.15	-0.1	0.05
rs62463305	-0.15	-0.1	0.04
rs28407053	-0.13	-0.61	-0.09
rs10250481	-0.13	-0.61	-0.08
rs62463306	-0.15	-0.01	0.05
rs6954646	-0.15	-0.01	0.05
rs10278735	-0.13	0.03	0.06
rs4723706	-0.14	0.08	0.16
rs4142516	-0.14	0.08	0.16
rs760694845	-0.15	0.03	0.04
rs2392520	-0.15	0.03	0.04
rs2392519	-0.15	0.02	0.04
rs73121244	-0.15	0.03	0.04
rs4720260	-0.15	0.03	0.04
rs10242196	-0.15	0.05	0.14
rs10245272	-0.15	0.05	0.14
rs10249901	-0.15	0.05	0.14
rs10257984	-0.15	0.06	0.11
rs17825495	-0.15	0.05	0.14
rs147171986	-0.15	0.05	0.14
7:37834268	-0.15	0.03	0.04
rs780986521	-0.15	0.03	0.02
rs12539577	-0.15	0.03	0.02
7:37837731	-0.15	0.03	0.02
rs2709110	0.15	-0.1	0.37
rs2718059	0.15	-0.1	0.37
rs10234857	-0.15	0.03	0.04
rs62464362	-0.15	0.03	0.04
rs201375814	-0.15	0.02	0.04
rs779355157	-0.15	0.03	0.04
7:37849687	-0.15	0.04	0.05
7:37850005	-0.15	0.03	0.04
rs1986714	-0.15	0.03	0.04
rs28867163	-0.15	0.03	0.04
rs28858459	-0.15	0.03	0.04
rs10241576	-0.15	0.03	0.04
rs1450847	0.15	-0.09	0.37

© 2024 Hoang T et al. *JAMA Network Open.*

Variant	Factor 1	Factor 2	Factor 3
rs35202238	0.15	-0.09	0.34
rs1597550	0.15	-0.09	0.34
rs2392516	-0.15	0.03	0.04
rs62459519	-0.15	0.03	0.04
rs1376256	0.15	-0.1	0.37
rs13437867	-0.15	0.03	0.04
rs2122624	-0.14	-0.35	-0.05
rs1530819	-0.15	0.02	0.04

© 2024 Hoang T et al. *JAMA Network Open.*

eTable 5. Associations Between Fish Intake and Colorectal Cancer Risk According to *EPDR1* Gene Pattern Scores

**Model**	**Fish intake (times/ week)**	**Low score (N=190,154)**		**High score (N=140,497)**	
		**No. cases**	**HR (95% CI)**	**No. cases**	**HR (95% CI)**
Crude	≤1	551	1.00 (ref.)	427	1.00 (ref.)
	>1 to ≤2	1,108	0.995 (0.898-1.103)	887	1.023 (0.916-1.155)
	>2	686	0.979 (0.875-1.096)	462	**0.852 (0.746-0.972)**
Sex-adjusted	≤1	551	1.00 (ref.)	427	1.00 (ref.)
	>1 to ≤2	1,108	1.007 (0.909-1.116)	887	1.043 (0.923-1.171)
	>2	686	0.999 (0.892-1.118)	462	**0.875 (0.766-0.998)**
Fully adjusted	≤1	551	1.00 (ref.)	427	1.00 (ref.)
	>1 to ≤2	1,108	1.056 (0.939-1.187)	887	1.083 (0.950-1.234)
	>2	686	1.023 (0.903-1.107)	462	**0.851 (0.732-0.990)**

HR, hazard ratio; CI, confidence interval. Fully adjusted model included sex, smoking, drinking, body mass index, and physical activity as covariates. Bold font indicates significant difference.

© 2024 Hoang T et al. *JAMA Network Open.*

eTable 6. Dietary Intake Among Study Among Participants Who Completed Touchscreen Questionnaire at Least Two Visit Assessments

Dietary factor	Baseline	Latest visit	P-value
**Red meat (times/week)**			
<2	892 (47.8)	959 (51.4)	**<0.001**
2 to <3	537 (28.8)	550 (29.5)	
≥3	438 (23.5)	358 (19.2)	
**Processed meat (times/week)**			
<1	682 (36.5)	729 (39.0)	0.15
1	549 (29.4)	515 (27.6)	
≥2	636 (34.1)	623 (33.4)	
**Poultry (times/week)**			
<1	301 (16.1)	327 (17.5)	**0.002**
1	708 (37.9)	635 (34.0)	
≥2	858 (46.0)	905 (48.5)	
**Total fish (times/week)**			
≤1	419 (22.4)	366 (19.6)	**<0.001**
>1 to ≤2	920 (49.3)	877 (47.0)	
>2	528 (28.3)	624 (33.4)	
**Milk (100 mL/day)**			
<2	579 (31.0)	634 (34.0)	**<0.001**
2 to <3	786 (42.1)	802 (43.0)	
≥3	502 (26.9)	431 (23.1)	
**Cheese (times/week)**			
<2	663 (35.5)	604 (32.4)	**0.004**
2 to 4	912 (48.8)	924 (49.5)	
≥5	292 (15.6)	339 (18.2)	
**Total fruit (servings/day)**			
<2	570 (30.5)	585 (31.3)	0.18
2 to <4	845 (45.3)	869 (46.5)	
≥4	452 (24.2)	413 (22.1)	
**Total vegetables (servings/day)**			
<4	665 (35.6)	666 (35.7)	0.73
<6	694 (37.2)	670 (35.9)	
≥6	508 (27.2)	531 (28.4)	
**Coffee (cups/day)**			
<1	474 (25.4)	421 (22.5)	**<0.001**
1 to ≤2	745 (39.9)	835 (44.7)	
>2	648 (34.7)	611 (32.7)	
**Tea (cups/day)**			

© 2024 Hoang T et al. *JAMA Network Open.*

Dietary factor	Baseline	Latest visit	P-value
<3	705 (37.8)	716 (38.4)	0.12
<5	555 (29.7)	586 (31.4)	
≥5	607 (32.5)	565 (30.3)	
**Alcohol (times/week)**			
<1	398 (21.3)	493 (26.4)	**<0.001**
1 to 2	442 (23.7)	454 (24.3)	
≥3	1,027 (55.0)	920 (49.3)	

Data are presented as counts (percentages). Differences of dietary intake between baseline and latest visit were analyzed using McNemar test.

© 2024 Hoang T et al. *JAMA Network Open.*

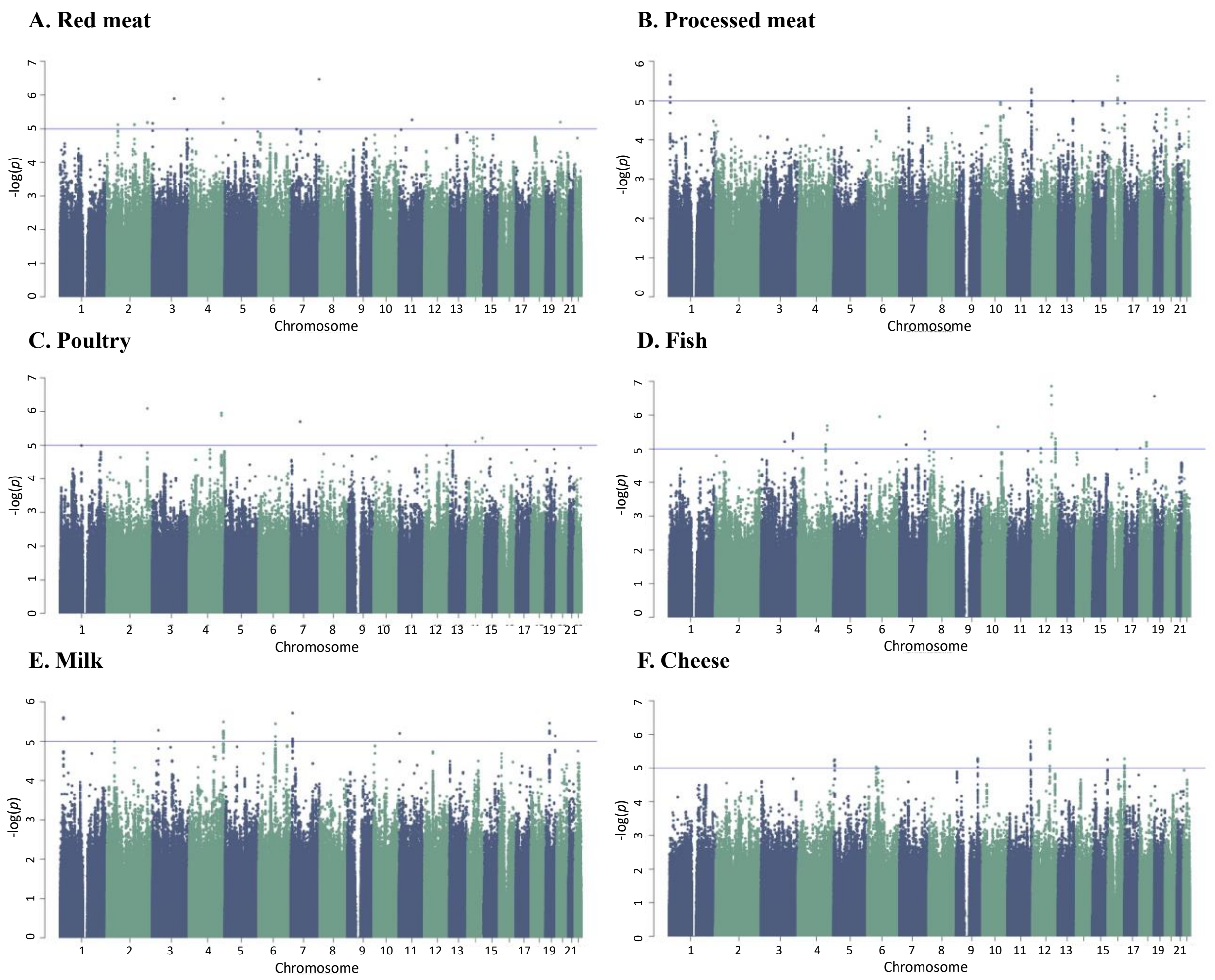

eFigure 1. Manhattan Plot of SNP-Diet Interactions Identified in the Genome-Wide Interaction Analysis of (A) Red Meat, (B) Processed Meat, (C) Poultry, (D) Fish, (E) Milk, and (F) Cheese

© 2024 Hoang T et al. *JAMA Network Open*.

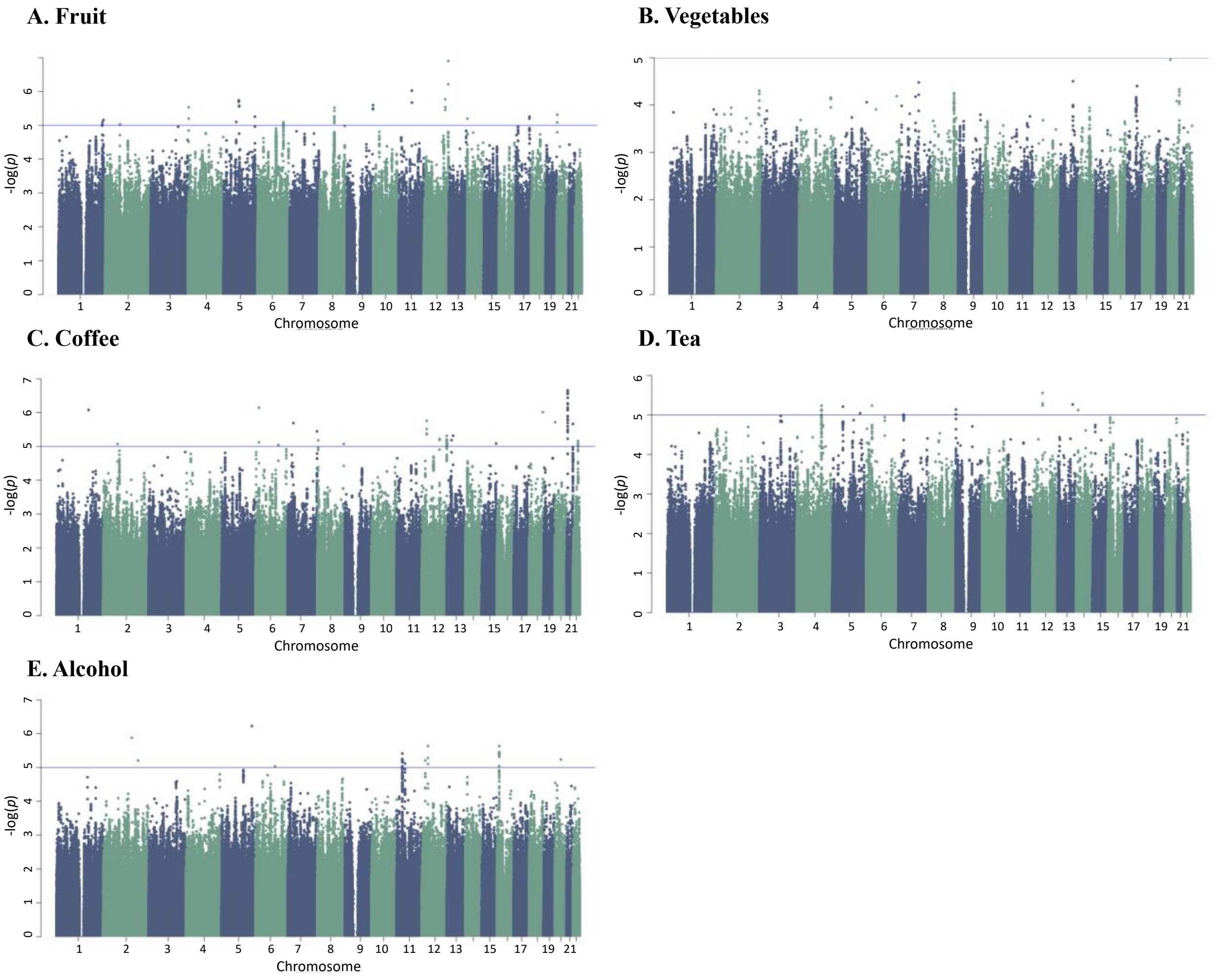

eFigure 2. Manhattan Plot of SNP-Diet Interactions Identified in the Genome-Wide Interaction Analysis of (A) Fruit, (B) Vegetables, (C) Coffee, (D) Tea, and (E) Alcohol

© 2024 Hoang T et al. *JAMA Network Open.*

Molecular functions

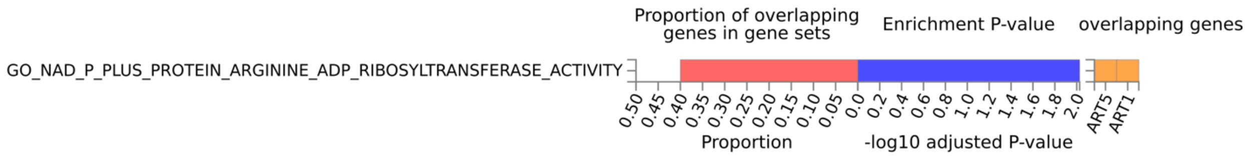

eFigure 3. Significantly Enriched Gene-Sets Interacted With Milk Consumption

© 2024 Hoang T et al. *JAMA Network Open.*

Biological processes

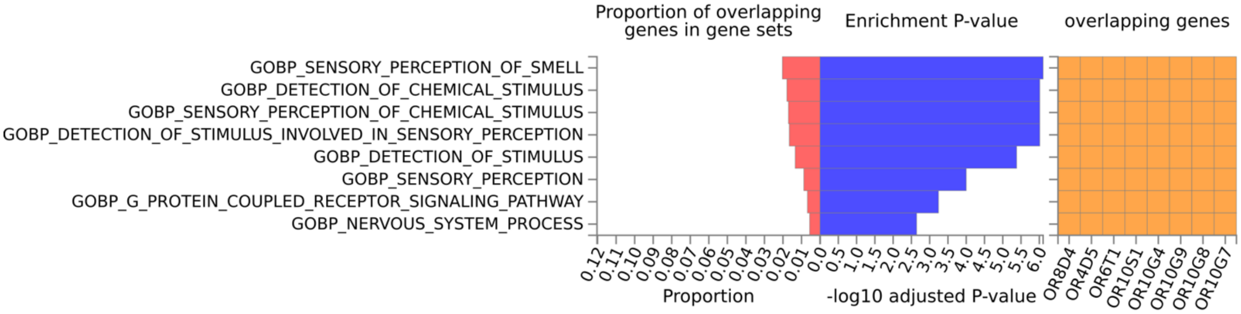

Molecular functions

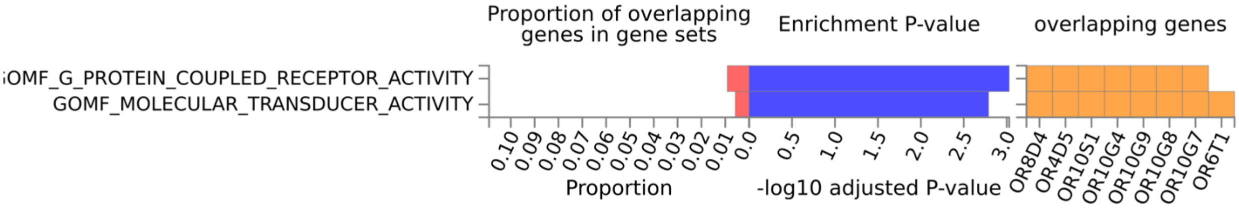

eFigure 4. Significantly Enriched Gene-Sets Interacted With Cheese Consumption

© 2024 Hoang T et al. *JAMA Network Open.*

Biological processes

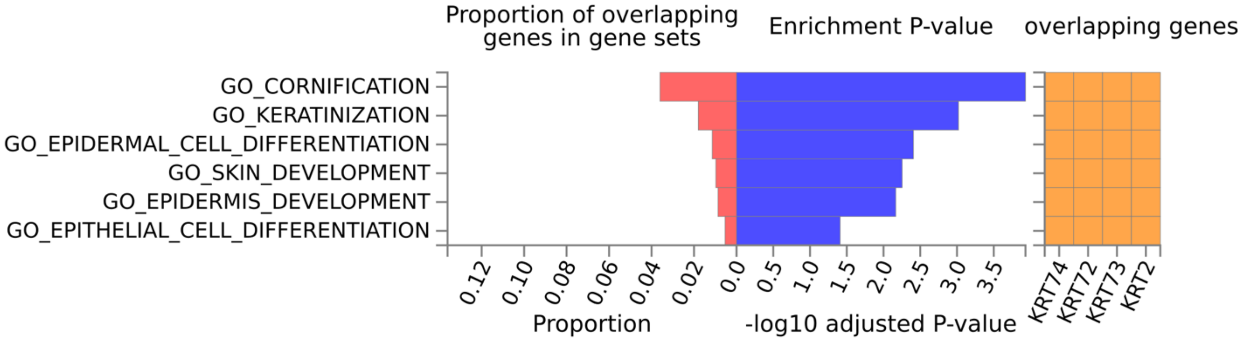

Cellular components

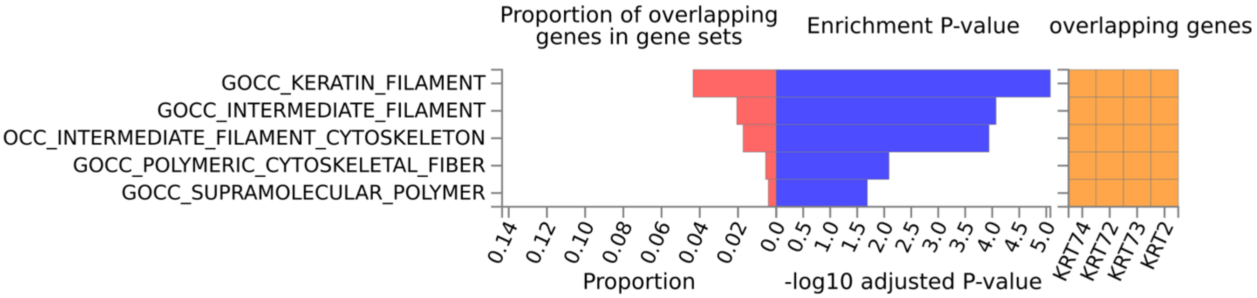

Molecular functions

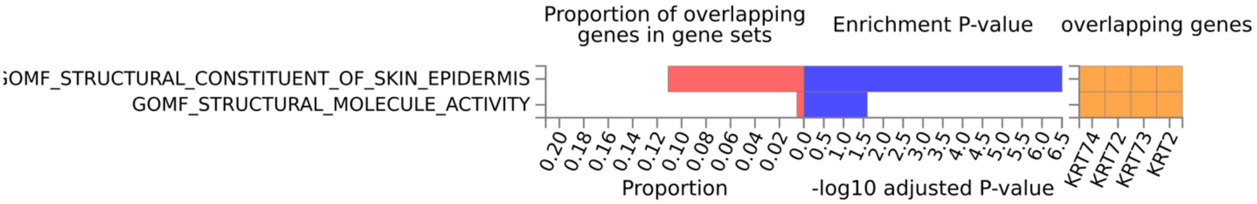

eFigure 5. Significantly Enriched Gene-Sets Interacted With Tea Consumption

© 2024 Hoang T et al. *JAMA Network Open.*

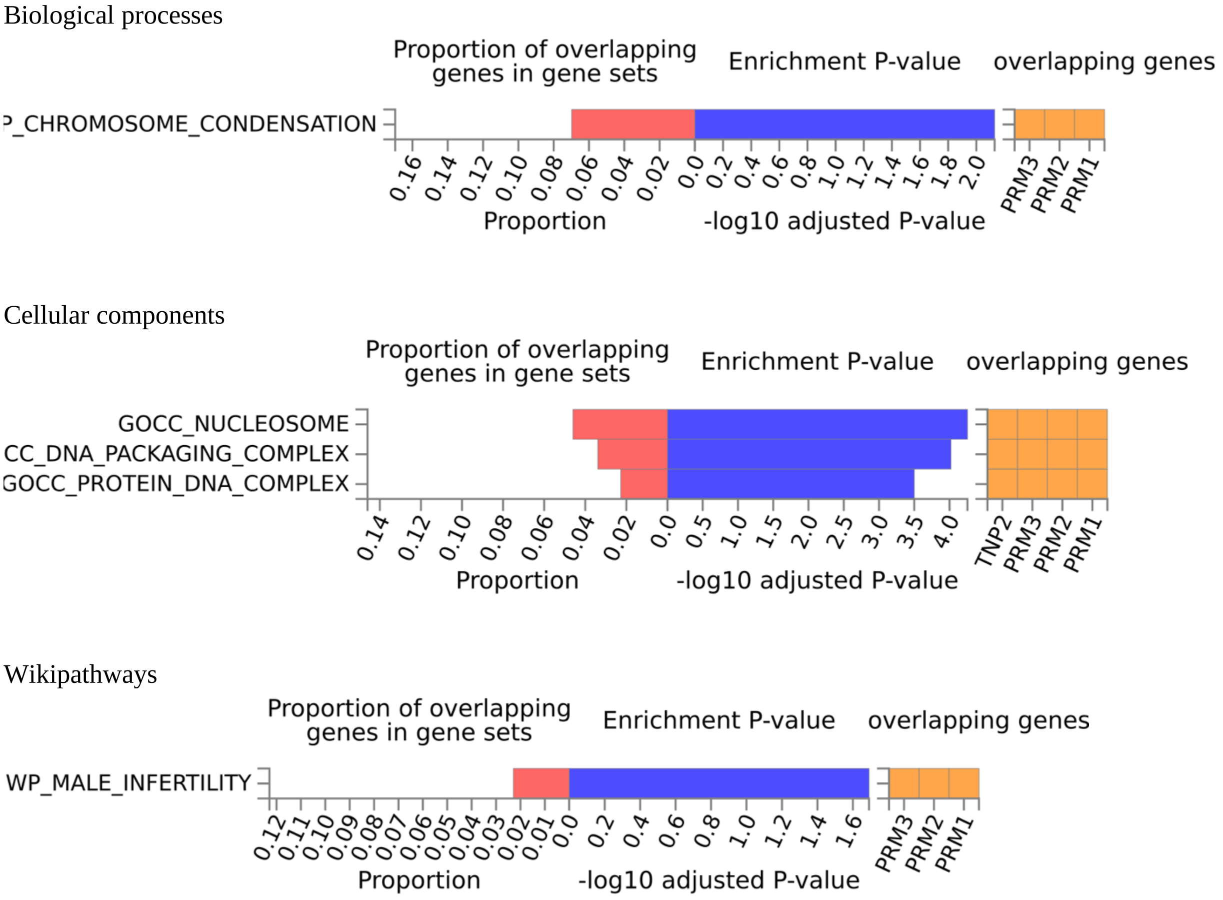

eFigure 6. Significantly Enriched Gene-Sets Interacted With Alcohol Consumption

© 2024 Hoang T et al. *JAMA Network Open.*

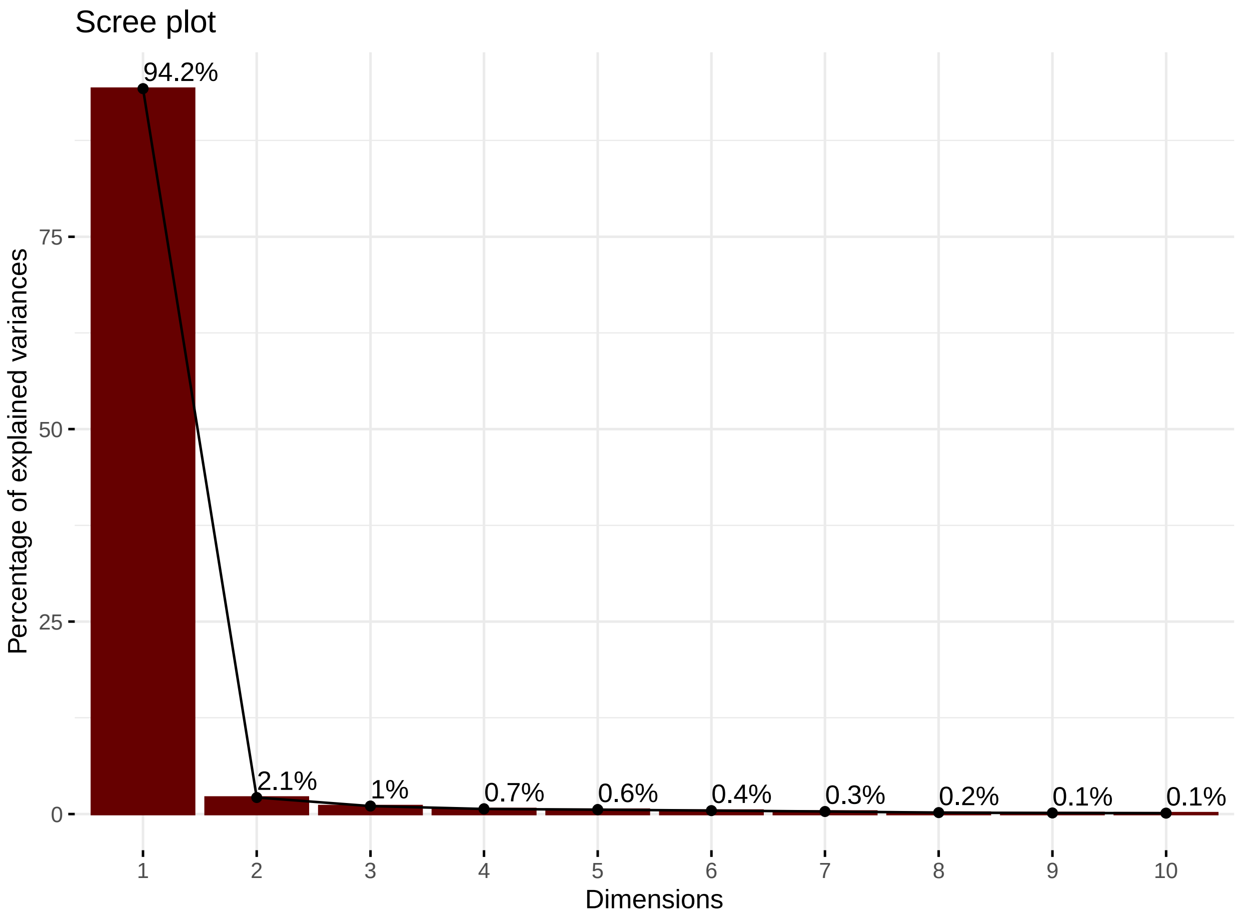

eFigure 7. Scree Plot for Explained Variance of Principal Components Derived From Multiple Variants Located in *EPDR1* Gene Using Principal Component Analysis

© 2024 Hoang T et al. *JAMA Network Open.*

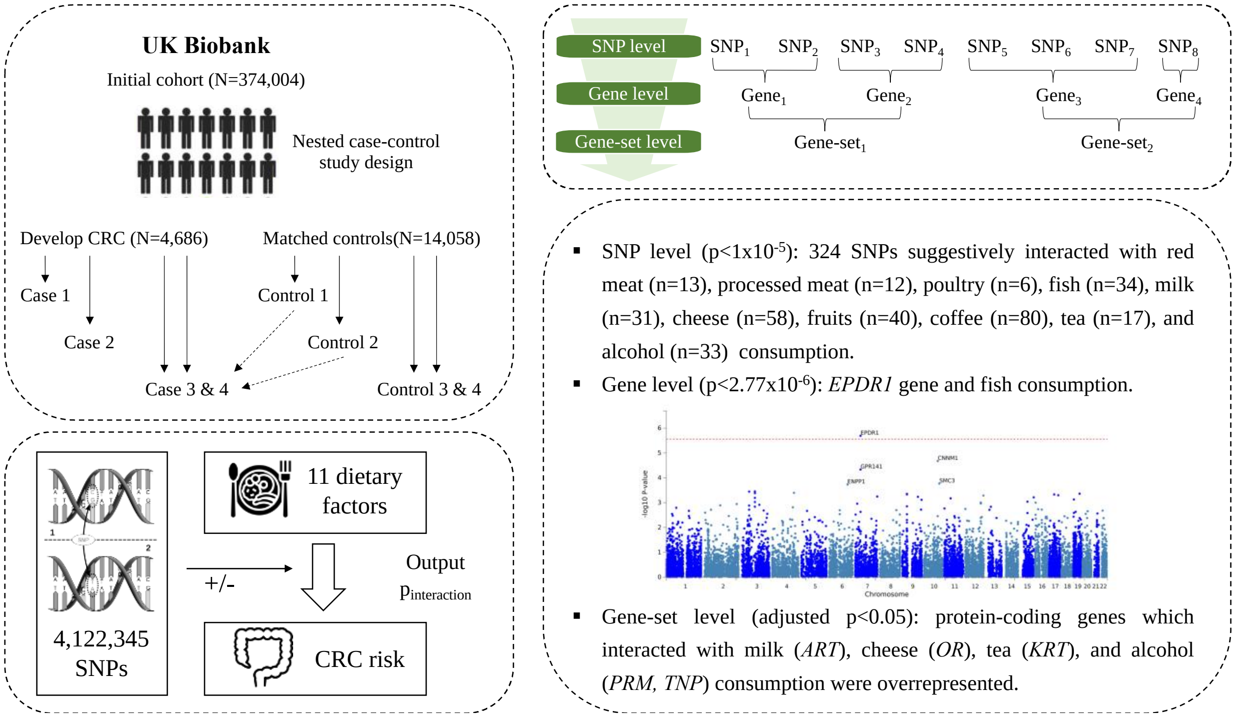

eFigure 8. Summary of Findings for Gene-Diet Interaction in Colorectal Cancer

© 2024 Hoang T et al. *JAMA Network Open.*
