# Supplementary material for: Genome-Wide Interaction Study of Dietary Intake and Colorectal Cancer Risk in the UK Biobank
Source: JAMA Netw Open. 2024 Feb 27;7(2):e240465. doi: 10.1001/jamanetworkopen.2024.0465 (PMC10900970; doi:10.1001/jamanetworkopen.2024.0465)
Supplement: Supplement 2. — Data Sharing Statement [file jamanetwopen-e240465-s002.pdf]

Data Sharing Statement

Hoang. Genome-Wide Interaction Study of Dietary Intake and Colorectal Cancer Risk in the UK Biobank. *JAMA Netw Open*. Published February 27, 2024.
doi:10.1001/jamanetworkopen.2024.0465

Data
Data available: No

Additional Information
Explanation for why data not available: The UK Biobank is an open access resource, available at https://www.ukbiobank.ac.uk/researchers/, and can be obtained from the UK Biobank by submitting a data request proposal. The data that support findings of this study were used under license for the current study (Application #94695), and so are not publicly available.
